# Structure and Function of a Fungal Adhesin that Binds Heparin and Mimics Thrombospondin-1 by Blocking T Cell Activation and Effector Function

**DOI:** 10.1371/journal.ppat.1003464

**Published:** 2013-07-11

**Authors:** T. Tristan Brandhorst, René Roy, Marcel Wüthrich, Som Nanjappa, Hanna Filutowicz, Kevin Galles, Marco Tonelli, Darrell R. McCaslin, Kenneth Satyshur, Bruce Klein

**Affiliations:** 1 Department of Pediatrics, University of Wisconsin School of Medicine and Public Health, Madison, Wisconsin, United States of America; 2 The Medical Scientist Training Program, University of Wisconsin School of Medicine and Public Health, Madison, Wisconsin, United States of America; 3 The Cell and Molecular Biology Graduate Training Program, College of Agriculture and Life Science, University of Wisconsin-Madison, Madison, Wisconsin, United States of America; 4 The Department of Biochemistry, The Biophysics Instrumentation Facility, College of Agriculture and Life Science, University of Wisconsin-Madison, Madison, Wisconsin, United States of America; 5 The Department of Bacteriology, The College of Agriculture and Life Science, University of Wisconsin-Madison, Madison, Wisconsin, United States of America; 6 Internal Medicine, University of Wisconsin School of Medicine and Public Health, Madison, Wisconsin, United States of America; 7 Medical Microbiology and Immunology, University of Wisconsin School of Medicine and Public Health, Madison, Wisconsin, United States of America; University of California, San Francisco, United States of America

## Abstract

*Blastomyces* adhesin-1 (BAD-1) is a 120-kD surface protein on *B. dermatitidis* yeast. We show here that BAD-1 contains 41 tandem repeats and that deleting even half of them impairs fungal pathogenicity. According to NMR, the repeats form tightly folded 17-amino acid loops constrained by a disulfide bond linking conserved cysteines. Each loop contains a highly conserved WxxWxxW motif found in thrombospondin-1 (TSP-1) type 1 heparin-binding repeats. BAD-1 binds heparin specifically and saturably, and is competitively inhibited by soluble heparin, but not related glycosaminoglycans. According to SPR analysis, the affinity of BAD-1 for heparin is 33 nM±14 nM. Putative heparin-binding motifs are found both at the N-terminus and within each tandem repeat loop. Like TSP-1, BAD-1 blocks activation of T cells in a manner requiring the heparan sulfate-modified surface molecule CD47, and impairs effector functions. The tandem repeats of BAD-1 thus confer pathogenicity, harbor motifs that bind heparin, and suppress T-cell activation via a CD47-dependent mechanism, mimicking mammalian TSP-1.

## Author Summary

Work on fungi is of worldwide importance due to the increasing burden of diseases caused by these agents in humans, plants and animals globally and throughout our ecosystem. The human pathogen *Blastomyces dermatitidis* harbors an essential virulence factor BAD-1. We describe new structural and functional features of BAD-1 that account for fungus' ability to cause disease. BAD-1 harbors a repetitive domain – a tandem repeat - that is present in up to 41 copies in the protein and essential in pathogenesis. We report an example of mimicry between this BAD-1 repeat and mammalian thrombospondin type-1 (TSP-1), a molecule that helps regulate human immunity. BAD-1 tandem repeats shares a tryptophan-rich sequence with TSP-1 that binds to tissue matrix and circulating immune cells. Like TSP-1, BAD-1 binds to this matrix. Through this property, BAD-1 binds and retards the action of white blood cells that fight fungal infection. Thus, we describe how fungi have evolved the means to mimic molecules that mammals commonly use to dampen unwanted immune responses, thus promoting pathogen survival.

## Introduction

The dimorphic fungus *Blastomyces dermatitidis* is endemic to the Ohio and Mississippi river valleys, where it is the causative agent of blastomycosis. Blastomycosis is one of the principal systemic mycoses of humans and animals worldwide, and results from the inhalation of spores and/or hyphal fragments released into the air by this soil-dwelling fungus. Pulmonary infections that go undiagnosed or untreated may progress and disseminate, leading to substantial morbidity and mortality even in immunocompetent hosts.

*Blastomyces* adhesin-1 (BAD-1) is a 120-kDa protein of *B. dermatitidis* that mediates multiple functions including adhesion, modulation of pro-inflammatory immune responses and virulence [1], [2], [3]. A targeted deletion of BAD-1 attenuates pathogenicity in a murine model of pulmonary infection. BAD-1 expression is yeast-phase specific and is induced during the temperature-driven morphological transition of *B. dermatitidis* mold to yeast [1], [4]. Following secretion, BAD-1 coats the yeast and mediates binding of yeast to macrophages by CD11b/CD18 (CR3) and CD14 receptors [1]. This binding fosters entry into phagocytes [1], while inhibiting the release of pro-inflammatory cytokines such as TNF-α [2], [3]. Surface bound BAD-1 inhibits TNF-α release in a TGF-β dependent manner, while secreted BAD-1 does so in a manner that is independent of TGF-β [5].

BAD-1 is composed of a short N-terminal region that harbors a secretion signal, an extensive core of tandem repeats that are responsible for adhesion [6], and a C-terminal EGF-like domain that anchors the released protein on the yeast surface by binding chitin [7]. The number of tandem repeats varies between *B. dermatitidis* strains [8], [9], but they typically comprise over 80% of the protein's primary sequence. The repeats share 20 strongly conserved amino acids, including two cysteines postulated to define a loop structure via disulfide bonding [10]. The tandem repeats bind divalent cations including calcium, zinc and copper [10] (1∶1 stoichiometry) and calcium binding enables the C-terminal EGF domain to fasten itself to exposed yeast cell-wall chitin. The binding of divalent cations and sequence similarities to EF-hand domains present in thrombospondin-1 (TSP-1), which is a multi-functional extracellular matrix protein [10], [11], previously lead us to postulate that the BAD-1 tandem repeats might coordinate divalent cations, triggering a conformational shift [10]. While we observed changes in the peptide mapping patterns of BAD-1 in the presence of calcium, elevated divalent cations unexpectedly failed to impact the secondary structure of the molecule as measured by circular dichroism and tryptophan fluorescence spectroscopy [10]. Those findings prompted questions about the native structure of BAD-1.

We sought here to elucidate the 3-D structure of BAD-1 by NMR to gain deeper insight into the function of its tandem repeats in the pathogenesis of *B. dermatitidis* infection. NMR structural analysis demonstrated that the repeats adopt a tightly folded 17-amino acid loop conformation, constrained by a disulfide bond between conserved cysteines. We found no evidence for a conformational shift upon interaction of the tandem repeats with divalent cations, nor evidence for an EF-hand structure. Rather, each tandem repeat loop contains a conserved WxxWxxW motif found in TSP-1 type 1 heparin-binding repeats. BAD-1 was confirmed to bind to heparin specifically, saturably and with high affinity. A novel BAD-1 action involved TSP-1-like suppression of T lymphocyte activation and effector function in a manner similarly dependent on heparan sulfate decoration of CD47 on T cells. Our work sheds new light on the structure and TSP-1-like function of BAD-1 in virulence, and offers a striking example of molecular mimicry likely contributing to the pathogenesis of this fungal disease.

## Results

### Primary structure of BAD-1

**Figure 1:**
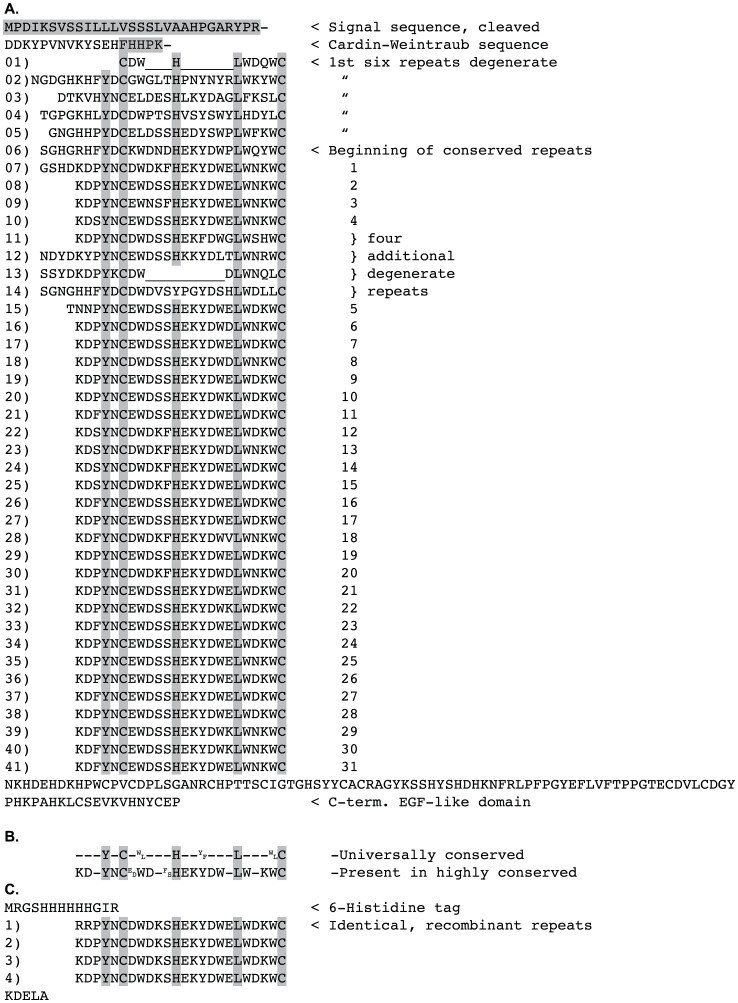
Primary structure of BAD-1 tandem repeats. **(A)** BAD-1 includes 31 highly conserved tandem repeats and 10 degenerate repeats, which make up 90% of the mature protein excepting the C-terminal EGF-like domain (103 amino acids) and 14 amino acids at the N-terminus. Universally conserved amino acids are highlighted. **(B)** Consensus of both universally conserved and highly conserved amino acids. **(C)** Sequence of the recombinant TR4 protein containing 4 identical repeats. Residues at each position represent the residues most commonly found in the corresponding positions of the native repeats.

Our earlier descriptions of BAD-1 in *B. dermatitidis* ATCC strain 26199 identified 30 tandem repeats based on their adherence to a consensus sequence [6]. This criterion may have been unduly stringent. Reanalysis of conserved sequences in BAD-1 shows that the tandem repeat domain extends to within 17 residues of the N-terminus of the mature protein (Fig. 1A). This analysis identifies 41 tandem repeats based on conservation of the distance between cysteine pairs and the presence of strictly conserved histidine, tyrosine, and leucine residues (Fig. 1B). This contrasts with prior characterizations of the N-terminus, and emphasizes the extent to which the tandem repeats dominate the primary structure of the protein. Thus, BAD-1 may be characterized as having two principal domains: an exceptionally long tandem repeat domain and a chitin-binding, C-terminal EGF-like domain that fixes it to the yeast cell surface.

### The tandem repeat mediates virulence

Since BAD-1 is an extended series of 41 tandem repeats with a short EGF-like C-terminal domain (Fig. 1), and since the C-terminal domain has proven to be dispensable for pathogenicity [12], we formally tested the role of the tandem repeats in virulence. To do so, we engineered a recombinant form of BAD-1 harboring only half the normal complement of tandem repeats and expressed this construct in a strain of *B. dermatitidis* (ATCC 26199) from which native BAD-1 had been deleted. Two independently engineered strains (TrepeatΔ20-Y and TrepeatΔ20-AE) were selected for their capacity to display amounts of surface BAD-1 similar to strains expressing the full-length protein (BAD1-6H-J and -AC) (Fig. S1). Each of these strains was compared in parallel for pathogenicity in a murine model of lethal pulmonary blastomycosis.

**Figure 2:**
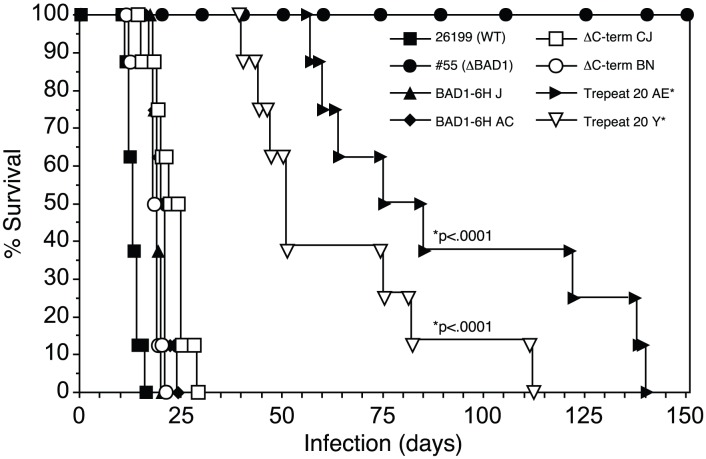
Role of BAD-1 tandem repeats in virulence of *B. dermatitidis in vivo*. Mice received various strains of engineered *B. dermatitidis* yeasts (10^4^) intra-nasally in 25-µl PBS. Strains included wild-type (ATCC 26199), isogenic BAD-1 knockout (#55), strain #55 transformed to re-express BAD-1 (BAD-1-6H) and truncated forms of BAD-1 lacking 20 repeats (Trepeat20) or the C-terminus (ΔC-term). Mice infected with yeast expressing Trepeat20 showed significantly increased survival compared to controls. Mice receiving recombinant strains expressing the full complement of tandem repeats showed no significant alteration in survival compared to mice receiving wild-type 26199 yeast. All mice receiving strain #55 survived until the experiment was terminated five months post-infection.

The transformed strains expressing the truncated forms of BAD-1 (TrepeatΔ20) were significantly less virulent than each strain expressing BAD-1 with the full complement of 41 repeats (Fig. 2). In contrast, the presence or absence of the C-terminal region (bearing all 41 repeats but no EGF-like C-terminal domain) had no significant impact on virulence in this model of infection as previously reported [12]. Thus, the tandem repeats of BAD-1 are required for pathogenicity in a murine model of lethal pulmonary infection.

### Structure of the tandem repeat

Because of the functional significance of the tandem repeats, we sought structural insight into these domains in full length BAD-1 via NMR. The large size of the protein and peak overlap resulting from minor sequence variations between repeats made it difficult to elucidate the 3-D structure of the full-length protein. We therefore expressed a set of representative tandem repeats of identical sequence in *E. coli* (their sequence reflects the most prevalent amino acid in each position). The shortest recombinant protein that we could express in quantity contained four tandem repeats (TR4). Initially, TR4 displayed a random assortment of disulfide linkages, determined by variations in mobility by non-reducing PAGE (Fig. S2). To correct this, TR4 was reduced, associated with an NiNTA column and then slowly refolded under a glutathione gradient (see Methods). After refolding, variations in mobility resolved into a single, predominant band (Fig. S2). Tryptic digests of TR4 in both refolded and reduced states were examined by LC-MS. Digests of refolded TR4 lacked the reduced versions of cysteine-containing peptides, confirming that the cysteines in TR4 are fully disulfide-linked. This was corroborated in NMR studies (below).

**Figure 3:**
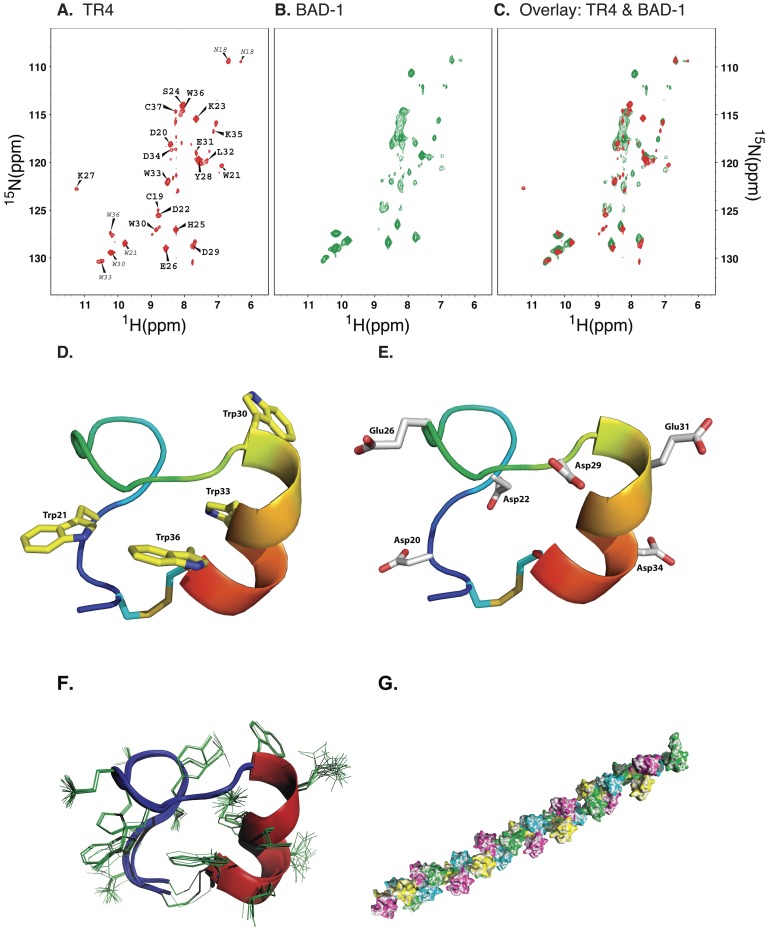
NMR determination of the structure of the tandem repeats. **(A)** ^1^H-^15^N HSQC spectrum of TR4. Peaks arising from backbone amides of the representative repeat are labeled, while peaks from side chain N-H groups are indicated with smaller labels. Note that the four tryptophan side chains yield four distinctive peaks indicating distinct chemical environments. **(B)** ^1^H-^15^N HSQC spectrum of full length BAD-1 protein. **(C)** ^1^H-^15^N HSQC spectra of TR4 and BAD-1 overlaid to show that the chemical shifts for the amino acids of the TR4 repeats are similar to those of the full length BAD-1 repeats. **(D)** Structure of one tandem repeat loop and its tryptophan residues. We determined the structure of one repeat, focusing upon the loop created by the disulfide bond between the two universally conserved cysteines. The repeat forms two tightly folded turns followed by a short α-helix. All but one of the tryptophan residues are buried in the center of the tandem repeat fold. **(E)** Structure of one tandem repeat loop and its acidic residues. Negatively charged residues are uniformly oriented on the external surface of the loop. **(F)** Overlapping image of the top 20 structural predictions by CYANA. Variability is seen primarily in externalized side-chains (depicted using thin green lines). **(G)** Theoretical structure of the BAD-1 molecule. Model is based on energy minimization of the hinge regions and the fact that extensive steric hindrance between the tandem-repeat loops further limits flexibility. In this model, tandem repeats lie along a helical twist, with roughly 3.2 repeats describing one full turn. Individual tandem repeat structures are sequentially colored: pink, yellow, green, blue (and repeated).

^15^N HSQC NMR analysis of this refolded TR4 molecule produced a pattern of peaks amenable to interpretation, but which otherwise corresponded closely to the pattern of peaks derived from ^15^N HSQC of full-length, native BAD-1 (Fig. 3 A–C). Thus the tandem repeats in the TR4 recombinant protein successfully replicate the conformation of the native repeats, and that these repeats predominantly adopt one uniform conformation. Alternative conformations, if present, must be minimally represented, rendering their NMR signature(s) undetectable.

Many residues of the four identical repeats in TR4 have very similar chemical shifts, resulting in peaks that closely overlap in their NMR spectra, but the spectra did vary slightly between repeats. Furthermore, no evidence of interaction between repeat domains was found in the NMR spectra and the hinge regions between the tandem repeats were not resolvable via NMR (probably attributable to localized flexibility). These results suggest that TR4 does not adopt a unique tertiary fold in solution and precluded resolution of the molecule as a single homogenous structure. As an alternative, we calculated the 3-D structure for a single, representative BAD-1 repeat. To this end, the NOESY data derived from the residues of one tandem repeat were identified, compiled separately and submitted for algorithmic derivation of distance constraints and structural calculation. To accommodate inconsistencies in NOE peak intensities derived from partially overlapping peaks, NOE-derived distance restraints (used for automatic calibration by CYANA) were relaxed, marginally increasing average distance limits. Nevertheless, this approach yielded a consistent, tightly-folded 17 amino acid loop structure constrained at the base by a disulfide bond between the two conserved cysteines (Fig. 3D). The only identifiable secondary structure within this loop was in the WxxWxxW motif, which forms a short α-helix. Constraints are reported in Table 1.

**Table 1:** Statistics of NMR Structures of TR4.

Conformationally restricting distance constraints	
Intraresidue [i = j]	69
Sequential [(i−j) = 1]	135
Medium Range [1<(i−j)≤5]	136
Long Range [(i−j)>5]	57
Total	397
Dihedral angle constraints	
φ	13
ψ	13
Number of constraints per residue	21.1
Number of long-range constraints per residue	2.9
CYANA target function [Å]	1.23±0.05
Average r.m.s.d. to the mean cyana coordinates [Å]	
backbone heavy (N18-C37)	0.40±0.06
all heavy atoms (N18-C37)	0.58±0.05
PROCHECK raw score (φ and Ψ/all dihedral angles) [69]	−0.84/−1.30
PROCHECK Z-scores (φ and Ψ/all dihedral angles)	−2.99/−7.69
MOLPROBITY raw score/Z-score [70]	27.35/−3.17
Ramachandran plot summary ordered residue ranges [%]	
most favored regions	85.6
additionally allowed regions	8.9
generously allowed regions	5.6
disallowed regions	0.0
Average number of distance constraints violations per CYANA conformer [Å]	
0.2–0.5	1.5
>0.5	0
Average number of dihedral-angle constraint violations per CYANA conformer [degrees]	
>10	0

The 3-D structure determined for the tandem repeat is inconsistent with the calcium-binding EF-hand structure previously hypothesized [10]. Every acidic residue is found on the external surface of the tandem repeat loop structure and not proximal to one another (Fig. 3E). Furthermore, the interior of the loop is occupied by aromatic residue side chains leaving little room for the pentagonal-bipyramidal calcium-coordination structure typical of an EF-hand. Thus, the high-capacity, low-affinity calcium-binding function of BAD-1 is not derived from an EF-hand-like structure in the tandem repeats. Alternatively, individual repeats could offer bi-dentate interactions with calcium, such that coordination of ions between repeat domains remains a possibility.

Figure 3F is a composite of the twenty best predictions available from CYANA and illustrates the consistency of this model with regard to the structure of the tandem repeat loop. Variability is predominantly seen in the orientation of surface-exposed side-chains interacting with the solvent environment.

### Predicted structure of full length BAD-1

Dynamic light scattering (DLS) data from two independent samples of BAD-1 showed polydispersity, but a relatively homogeneous population with a hydrodynamic radius of 7.2±0.5 nm accounted for ∼70% of the scattering intensity. This hydrodynamic radius contains contributions from the protein, bound water of hydration and shape factors (frictional coefficients) [13]. From the partial specific volume of BAD-1, the unhydrated molecule would have a spherical radius of 3.4 nm, which increases to 4.1 nm upon hydration. These values are much smaller than those measured by DLS. It would require a sphere comprised of 6 hydrated BAD-1 polypeptides as the diffusing complex to achieve the measured hydrodynamic radius. Sedimentation equilibrium studies showed BAD-1 to be monomeric. BAD-1 did not tend to oligomerize (up to at least 1.5 µM), suggesting that the large hydrodynamic radius is not due to formation of oligomers. We thus attribute the large measured radius to asymmetry in the shape of the molecule. The ratio of the measured radius to that of the sphere composed of a single hydrated BAD-1 molecule provides a quantitative estimate for this asymmetry, 1.8. The simplest models used to interpret hydrodynamic shapes are based on prolate and oblate ellipsoids of revolution. For an asymmetry of 1.8, a prolate ellipsoid would have semiaxes in the ratio of 15∶1∶1; an oblate ellipsoid would have axes in a ratio of 20∶20∶1. Our measurements suggest that the native conformation of BAD-1 occupies an expanded space - either a semi-flexible chain adopting multiple configurations or an extended rod-like structure.

NMR data suggests a 5 amino-acid flexible “hinge” between each tandem repeat “loop”, like beads on a string. Given the results of DLS, it is likely that BAD-1 is elongated with little in the way of tertiary structure. The hinge regions would afford limited flexibility. Energy minimization of the hinge regions of this model, constrained by the steric limitations of the tandem repeat “beads”, supports a (theoretical) extended, helical conformation (Fig. 3G).

### A tryptophan rich motif in the tandem repeat

Each tandem repeat loop of BAD-1 contains a significant number of tryptophans (4 of 24 amino acids - 17%). Because of this, BAD-1 absorbs UV wavelengths exceptionally well and may be detected readily by its characteristic OD280. A 1 mg/ml preparation of BAD-1 has an OD280 of over 6.6 [14]. The conserved arrangement of the tryptophans likewise stands out. With rare exception, three of the four tryptophans are arranged in a WxxWxxW motif. This motif is common to a number of glycosaminoglycan (GAG)-binding proteins, and is highly conserved within the type 1 heparin-binding repeats of TSP-1 [15].

**Figure 4:**
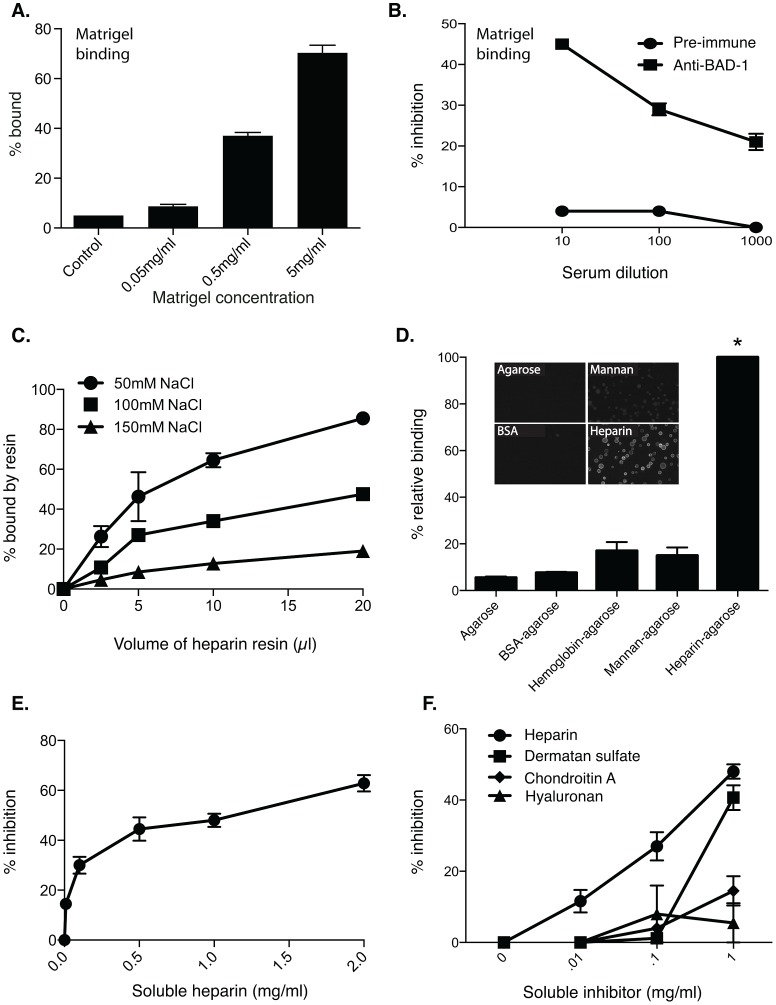
BAD-1 binding of heparin. **(A)** Percentage of 1×10^6^ *B. dermatitidis* yeast that bound to wells coated with increasing concentrations of Matrigel. Control contained no Matrigel. **(B)** Inhibition of yeast binding to Matrigel by different dilutions of anti-BAD-1 antiserum. **(C)** BAD-1 binding of heparin is saturable. 100 µl of 0.1 mg/ml BAD-1 was applied to various bed volumes of heparin-agarose resin (400–1500 ng heparin/µl resin) in the presence of 50 mM, 100 mM, and 150 mM NaCl. Unbound BAD-1 was quantified by A280. **(D)** Binding of BAD-1 to other resins. BAD-1 (eFluor605) pulled down with heparin agarose resin produced a robust fluorescent signal (rightmost column-positive binding control). Binding of BAD-1 (eFluor605) to uncoated agarose resin and resins coated with BSA, hemoglobin or mannan was measured for comparison. Fluorescent BAD-1 bound better to heparin agarose than each control (*, p<0.05), and binding to control resins was insignificant (p>0.05). Inset: Fluorescent BAD-1 binding to resin surfaces analyzed by fluorescence microscopy. **(E)** Inhibition of BAD-1 binding to heparin resin by soluble heparin. Fluorescent BAD-1 was pre-incubated with increasing concentrations of soluble heparin before exposure to heparin-agarose resin. **(F)** Inhibition of BAD-1 binding to heparin agarose by alternate GAGs. 0.1 mg/ml fluorescent BAD-1 was pre-incubated with heparin, dermatan sulfate, chondroitin sulfate A, or hyaluronan for 20 min, followed by incubation with heparin-agarose for 30 min. Inhibition by heparin is significant vs. controls and other GAGs. *, p<0.05. Chondroitin sulfate A and hyaluronan are not significantly different from each other or controls. Dermatan sulfate inhibits BAD-1 binding only at 1 mg/ml, but not at lower concentrations. Results are the mean ± SEM of two to five experiments/panel.

We initially tested whether BAD-1 might bind GAGs in the extracellular matrix (ECM) by studying the adherence of yeast to Matrigel, which contains heparan sulfate and other ECM proteins such as laminin, collagen IV and nidogen. *Blastomyces* yeast bound Matrigel (Fig. 4A). Anti-BAD-1 antiserum blocked yeast binding to Matrigel (Fig. 4B), suggesting that the binding is BAD-1 dependent. Yeast did not reproducibly bind to the highly purified ECM components laminin, collagen IV or nidogen alone, implying that other Matrigel constituents such as heparan sulfate proteoglycan might be a target of BAD-1.

### Heparin binding by BAD-1

We investigated whether native BAD-1 protein could bind heparin. Initially, purified BAD-1 was incubated with a heparin-coated agarose resin. Due to the strong UV absorbance of BAD-1, the percentage of BAD-1 that bound to resin could be assayed spectrophotometrically by measuring the OD280 of the aqueous phase before and after incubation. Heparin-agarose resin pulled BAD-1 (100 µl of 0.1 mg/ml) out of solution, with a 20 µl volume of resin absorbing nearly 100% of the soluble BAD-1. Binding was dependent upon resin volume (0.5–1.5 µg BAD-1/µl resin) and saturable (Fig. 4C). While binding was maximal at lower ionic strengths (≤50 mM NaCl), it was still appreciable at the established ionic strength of alveolar fluid (100 mM NaCl) and plasma (150 mM NaCl) [16], [17]. In subsequent assays, we fluorescently labeled BAD-1 (with eFluor605) to quantify binding via fluorescence spectroscopy. Labeling did not alter its binding characteristics since assays with unlabeled protein gave similar results (Fig. S3). Fluorescent BAD-1 bound avidly to heparin-agarose, but not to controls of unmodified agarose resin or resins coated with mannan, BSA or hemoglobin (Fig. 4D). We included the two latter controls since BAD-1 [10] and heparin [18] both coordinate polyvalent cations and have the potential for non-specific association via polyvalent cation bridging [19]. BSA [20] and hemoglobin [21] also coordinate multiple polyvalent cations. Nevertheless, BAD-1 showed little affinity for these control resins or for the carbohydrate-rich mannan-agarose resin.

We assessed the specificity of the interaction between BAD-1 and immobilized heparin using soluble competitors (Fig. 4E). Pre-incubation of BAD-1 with soluble heparin diminished its binding to agarose-immobilized heparin in a concentration-dependent manner. Closely related GAGs, including chondroitin sulfate A and hyaluronan, did not significantly inhibit the binding of BAD-1 to immobilized heparin (Fig. 4F). Dermatan sulfate, also called chondroitin sulfate B, inhibited only at the highest concentrations.

### Surface plasmon resonance measurements of the interaction of BAD-1 and heparin

**Figure 5:**
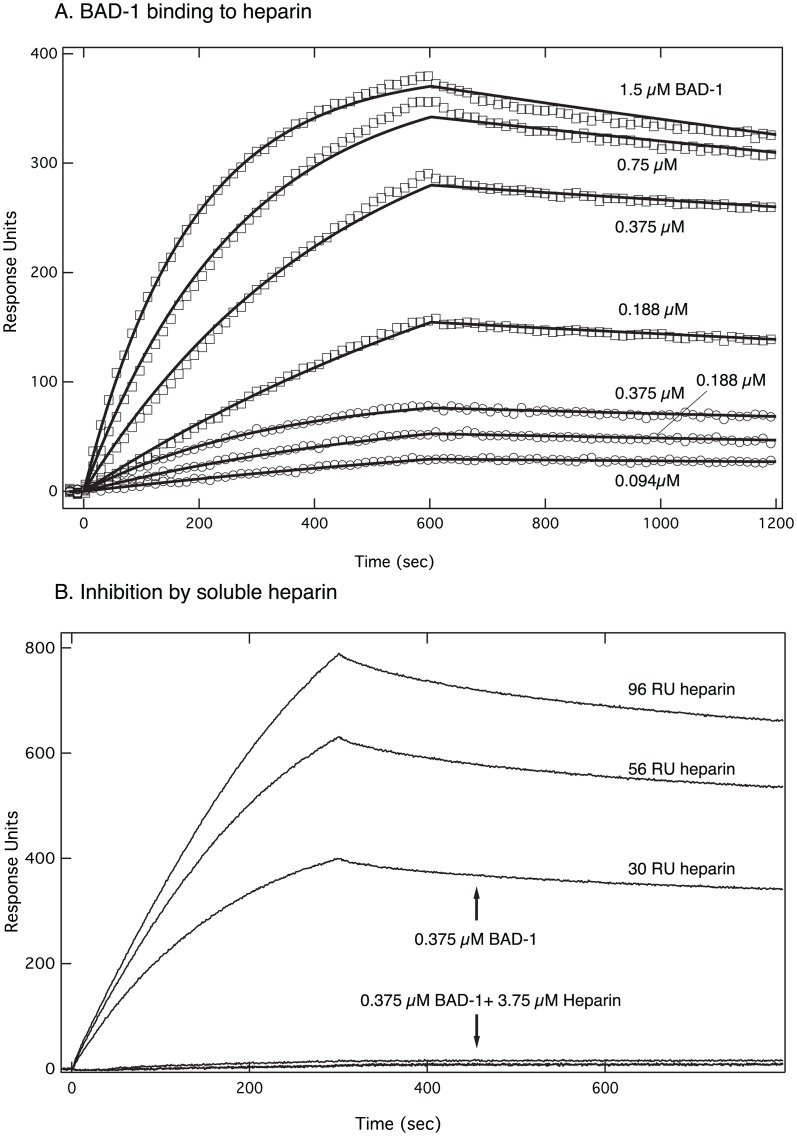
Affinity of BAD-1 for heparin measured by SPR. **(A)** BAD-1 binding to immobilized heparin monitored by surface plasmon resonance detection (SPR) using a Biorad Proteon XPR36. BAD-1 at the indicated concentrations was injected onto Biorad NLC neutravidin surface with biotinylated heparin immobilized to levels of 5 (circles) and 30 (squares) RUs. For clarity, only every 15th data point is shown. The solid lines are fits to the Langmuir binding model, on and off rates were fit to each sensogram but maximal response was fit to a single value for each immobilization level. The affinity was calculated from the rate constants to be 33±14 nM. **(B)** Heparin inhibition of BAD-1 binding to biotinylated heparin immobilized on a neutravidin surface. 0.375 µM BAD-1 with and without the addition of 3.75 µM heparin was injected onto the surface with heparin immobilized to levels of 30 (low), 59 (intermediate), and 96 (high) RU. Binding assays were performed using a buffer at physiological ionic strength (100 mM NaCl), similar to that in alveolar mucous (see Methods) [16], [17].

To measure the affinity of BAD-1 for heparin, biotinylated heparin was immobilized to several densities on a neutravidin NLC chip. Dose-response curves were generated with BAD-1 in concentrations ranging from 1.5 µM to 94 nM. Figure 5A shows the results for two low-density heparin surfaces. A striking feature is the slow dissociation of BAD-1 once it has bound to the surface. This likely reflects the multivalency of BAD-1. Figure 5B shows binding of 0.375 µM BAD-1 in the absence of heparin or mixed with 3.75 µM heparin. Binding to immobilized heparin is completely blocked by a 10-fold excess of free heparin. These two figures establish the specific binding of BAD-1 to the heparin surface.

The kinetic constants for the dose-response data above were calculated using the Langmuir 1∶1 binding model in the instrument software. For the curves shown in figure 5A, the on and off rates were fit to each sensorgram separately, but the maximum response level was fit as a single value for each heparin density. The on-rate was 5800±2000/M/s with an off-rate of 1.7±0.3 e-4/s. Each pair of rate constants was used to compute an equilibrium dissociation constant, or affinity, of 33±14 nM, which closely approximates that reported for TSP-1 (80 nM) [22]. Thus, BAD-1 binds to immobilized heparin in a concentration-dependent manner, which is inhibitable by free heparin. Once bound, BAD-1 dissociates from the heparin surface slowly, demonstrating the kind of high avidity/moderate affinity interaction typical of GAG-binding proteins with multiple binding sites [23].

SPR analysis of the binding of truncated Trepeat20 BAD-1 to heparin showed little variation in affinity compared to full length BAD-1 (Fig. S4). The reduced virulence observed for strains producing this truncated adhesin is thus not explained by a diminished affinity or avidity for heparin, but may hinge on some other factor(s). Nevertheless, reduced length is a feature known to impact the function of other adhesins [24]. Alternatively, if individual (free) BAD-1 repeats were to mediate *in vivo* effects on interaction with the immune system (below), the Trepeat20 strain has only half the molar equivalent of repeats of the parental strain.

### Characterization of the interaction between BAD-1 and heparin

The interaction between heparin and TSP-1 involves an ionic component and may be inhibited by concentrations of NaCl above 350 mM [22]. We observed that NaCl could similarly inhibit the binding of BAD-1 to heparin-agarose (Fig. S5A). Binding of BAD-1 to heparin diminished sharply at 250 mM NaCl and above, suggesting that this interaction involves an ionic component. The binding is maximal at low pH, but falls off substantially above pH 8 (Fig. S5B), indicating that protonated histidine residues may be integral to the binding site

**Figure 6:**
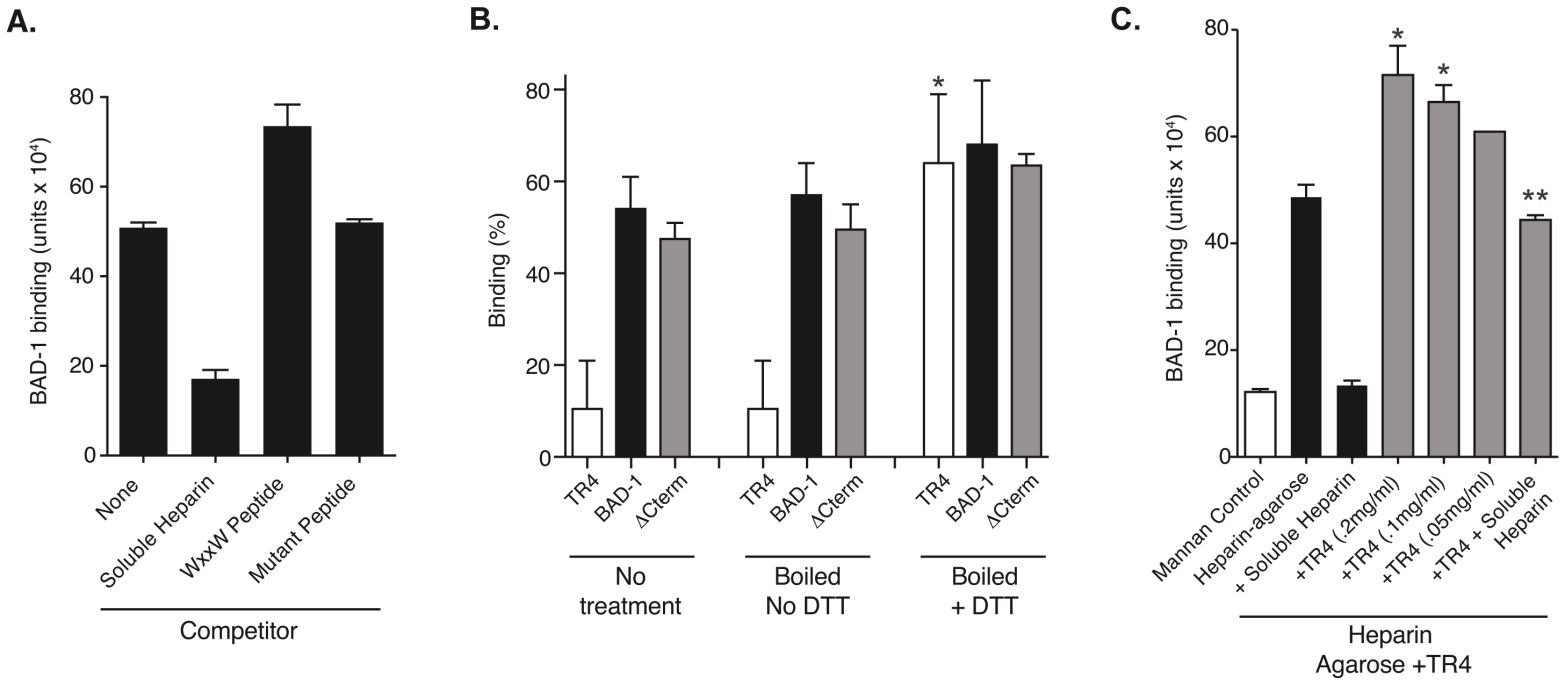
Influence of competitors on BAD-1 binding to heparin. **(A)** Effect of a WxxW motif heparin-binding peptide on binding of BAD-1 (eFluor605) to immobilized heparin. “None” denotes BAD-1 binding to heparin agarose with no competitor. The WxxW peptide, or a mutant control peptide, was incubated with heparin resin at 1 mg/ml before addition of fluorescent BAD-1. Binding was quantitated by fluorescence units detected in a Filtermax F5 plate reader. Results are the mean ± SEM two experiments. **(B)** Effect of TR4 reduction on binding to heparin. Samples were incubated with resin directly or first boiled for 3 min in buffer alone or buffer with 5 mM DTT. TR4 has four copies of the BAD-1 tandem repeat. ΔCterm has all 41 repeats, but no C-terminal EGF-like domain. Binding was quantified by A280 measurement. Reduced TR4 bound significantly better than untreated TR4 or TR4 boiled without DTT (*, p<0.05). Results are the mean ± SEM of two experiments. **(C)** Effect of reduced TR4 on binding of BAD-1 (eFluor605) to immobilized heparin. BAD-1 binding to heparin agarose was quantified with or without pretreatment of resin with reduced TR4 as in panel C. BAD-1 binding was quantified by fluorescence units as above. Mannan resin is a background control. Heparin resin pre-treated with reduced TR4 at 0.2 and 0.1 mg/ml bound BAD-1 significantly better than untreated heparin resin (*, p<0.05). Soluble heparin significantly blocked binding of BAD-1 to both of these pre-treated resins (**, p<0.05).

The interaction between TSP-1 and heparin is inhibited by peptides containing a WxxW motif [15]; for example a peptide with the sequence SHWSPWSS. We used this peptide (and a control, mutant peptide with tryptophan residues replaced by glutamine) to test whether BAD-1 binds to the same site on heparin. The WxxW peptide failed to inhibit, but instead augmented BAD-1 binding to heparin agarose (Fig. 6A), and control peptide (SHQSPQSS) had no effect on binding. Soluble heparin blocked 65% of BAD-1 binding to heparin agarose. Thus, the initial binding of BAD-1 to heparin could be facilitated by a site outside the tandem repeat (see below).

We also tested whether TR4 could be used to block BAD-1 binding to heparin agarose, as each repeat bears a WxxWxxW motif. Native TR4, however, bound poorly to immobilized heparin (Fig. 6B). BAD-1 lacking a C-terminal EGF domain (ΔC-term) did bind heparin, which implied that this domain does not mediate heparin binding. TR4 could only be induced to bind heparin by reducing the peptide's disulfide bonds with DTT and allowing re-oxidation in the presence of heparin. This maneuver also enhanced heparin binding by BAD-1 and ΔC-term protein, but when these proteins were maintained in a reduced state (DTT≥1 mM) the capacity to bind heparin was lost (data not shown), suggesting that primary sequence alone is insufficient to drive the heparin interaction and that a novel secondary structure must form to bind heparin.

Once we established that DTT-unfolded TR4 binds heparin agarose upon oxidation, we investigated whether this domain – harboring a WxxWxxW motif – could interfere with BAD-1 binding to heparin. As with the synthetic WxxW peptide, reduced TR4 failed to inhibit binding and instead augmented BAD-1 binding to heparin agarose in a concentration-dependent manner (Fig. 6C). This result suggests that the WxxWxxW motif in the tandem repeats is not enough, by itself, to foster initial interaction with heparin. An alternate possibility is that it may be half of the two-component heparin-binding cleft described by Cardin-Weintraub [25]. Thus, instead of inhibiting binding, tryptophan-containing peptides might pair with stretches of basic residues to stimulate binding. In fact, examination of the N-terminus of BAD-1 reveals an xBBxBx Cardin-Weintraub heparin-binding motif [26] (B = basic residue, x = any residue) within this short stretch of amino acids (Fig. 1A). This known heparin-binding motif could be involved in the initial engagement of heparin (or similar polysulfated GAG) by BAD-1.

### BAD-1 inhibits T cell activation via CD47

**Figure 7:**
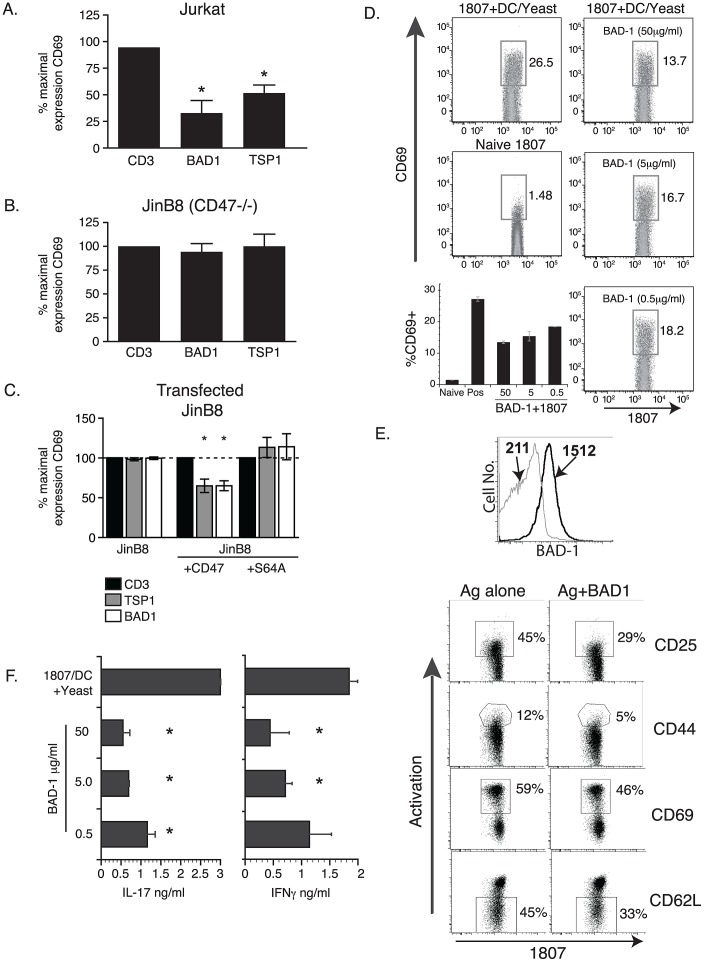
Suppression of T cell activation mediated by BAD-1 binding of CD47. **(A)** BAD-1 inhibition of CD69 expression is dependent on CD47. Jurkat T cells were activated by anti-CD3 antibody alone (5 µg/ml) or in the presence of BAD-1 or TSP1 (10 µg/ml) for 2 hours *in vitro*. RNA was isolated and relative CD69 mRNA expression determined by real-time PCR. **(B)** JinB8 T cells lacking CD47 were activated by anti-CD3 antibody alone or in the presence of BAD-1 or TSP1 for 2 hours *in vitro*. RNA was isolated and relative CD69 mRNA expression determined as above. **(C)** Un-transfected JinB8 cells and cells transfected with CD47 and CD47-S64A were activated by anti-CD3 antibody alone or in the presence of BAD-1 or TSP-1 for 2 hours *in* vitro. RNA was isolated and relative CD69 mRNA expression determined as above. Values are percent activation relative to stimulated cells ± SD of 4 experiments for data in panels A–C; *, p<0.05. **(D)** CD4^+^ T cells from 1807 TCR Tg mice were exposed to increasing amounts of BAD-1 for 90 minutes, washed and added to co-culture of BAD-1 null *B. dermatitidis* yeast and DC for 96 hours. After incubation, the T cells were analyzed by flow cytometry for expression of CD69. **(E)** BAD-1 (50 µg/ml) was incubated with 1807 cells at 37°C for 90 min and analyzed by FACS with anti-BAD-1 mAb conjugated to FITC. In top panel, the numbers are the mean fluorescence intensity of mAb binding to control-treated (grey) vs. BAD-1-treated (black) cells. After incubating 1807 Tg cells as above, the T cells were added to wells containing DC and *Blastomyces* cell wall/membrane antigen (10 µg/ml). After 24 hours the cells were stained for the activation markers shown and analyzed by FACS. **(F)** Supernates were collected from co-cultures in panel D and assayed by ELISA for IL-17A and IFN-γ. *, p<0.05. vs. control. Results are representative of 2–4 independent experiments for panels D and E.

Molecules with heparin-binding motifs can modulate the activation of immune cells such as T cells via interaction with GAG-modified cell surface proteins. The heparin-binding protein, TSP-1, inhibits the activation of T cells via interaction with the surface protein CD47 through a GAG-modified serine at position 64 [27]. While the C-terminal TSP-1 domain is sufficient for this interaction [28], we hypothesized that BAD-1 might also block T cell activation in a CD47 dependent manner. Anti-CD3 antibody activation of Jurkat T cells or CD47-deficient JinB8 T cells resulted in a ∼25-fold increase in CD69 expression at 2 hours. As reported previously [27], TSP-1 decreased CD69 expression in activated Jurkat T cells by 45% (Fig. 7A), while failing to inhibit the activation of JinB8 T cells (Fig. 7B). BAD-1 also sharply reduced CD69 expression in activated Jurkat T cells by 65%, but failed to block activation of CD47-deficient JinB8 T cells.

We next tested the role of GAG modification of CD47 in BAD-1 suppression of T cell activation. We transfected CD47-deficient JinB8 T cells with a plasmid encoding wild-type CD47 or a mutant CD47 containing a serine to alanine substitution that precludes GAG-modification (CD47-S64A). Transfections led to re-expression of CD47 on JinB8 cells. Re-expression of wild-type CD47, but not CD47-S64A, in JinB8 cells enabled TSP-1 to significantly inhibit CD69 induction by 35.1% (Fig. 7C). BAD-1 also significantly inhibited CD69 induction in JinB8 cells transfected with wild-type CD47 (34.9%), but not in JinB8 cell transfected with CD47-S64A. BAD-1, like TSP-1, thus modulates T cells activation via surface protein CD47 and the inhibitory action of BAD-1 requires CD47 GAG-modification at Serine 64.

We next tested the impact of BAD-1 on the function of primary CD4^+^ T cells that respond to *Blastomcyes* in an antigen-specific manner and mediate immunity during infection [29]. 1807 TCR transgenic mice produce such *Blastomyces* reactive CD4^+^ T cells [30]. We studied naïve CD4^+^ T cells from these mice, analyzing their ability to become activated and differentiate into cytokine producing cells *in vitro* in response to co-culture with BAD-1 null *Blastomyces* yeast and dendritic cells (DC). Upon culture with yeast, 1807 cells became activated as measured by CD69 expression in ∼30% of the cells (Fig. 7D). 1807 cells also responded to the fungus by producing IL-17 and IFN-γ (Fig. 7F). The addition of exogenous BAD-1 to 1807 cells curtailed their activation and expression of CD69 in response to co-culture with yeast and DC (Fig. 7D), and was associated with binding to the T cell surface (Fig. 7E). In addition to CD69, several other markers of T cell activation, including CD25 (IL-2R), CD44 and CD62L, showed that 1807 responses to antigen were similarly blunted by BAD-1 (Fig. 7E). BAD-1 also suppressed 1807 cell production of IL-17 and IFN-γ in a concentration dependent manner (Fig. 7F). Thus, BAD-1 mimics TSP-1 and is capable of suppressing activation and effector functions of T cells, in this case CD4^+^ T cells that confer resistance to infection.

## Discussion

Herein, we describe novel structural and functional properties of BAD-1, an essential virulence factor of *B. dermatitidis*. We establish that the tandem repeats are indispensible for the role of BAD-1 in virulence, provide NMR based 3-D structural characterization of the BAD-1 tandem repeats, and identify a tryptophan-rich motif in the tandem repeats involved in heparin binding. We further establish that BAD-1 suppresses T cell function via interaction with CD47, thus mimicking TSP-1. Though initial analyses of the primary structure of BAD-1 reported 30 highly conserved tandem repeats [6], a more in-depth assessment of stringently conserved elements adds 11 more repeats to this total. This new model characterizes BAD-1 as essentially an extended series of tandem repeats (comprising >80% of the protein's length) with a C-terminal, chitin-binding domain to anchor it to the surface of *B. dermatitidis* yeast and a predicted N-terminal Cardin-Weintraub heparin-binding site [26]. Because the C-terminus is dispensable for virulence in a murine model of pulmonary infection [12], we postulated that virulence must depend upon the tandem repeats. Indeed, we found that even partial deletion of the repeats attenuates pathogenicity mediated by BAD-1.

The tandem repeats of BAD-1 contribute to its adhesive functions [1], [6]. Pathogen surface adhesins often contain tandem repeat domains. In some cases, the tandem repeats themselves mediate host-cell surface adhesion (e.g. *Staphylococcus aureus* MSCRAMM) [31]. In other cases, the repeats act as “spacer arms” that orient and present a binding domain to host receptors. Upon engagement of host ligands, certain adhesins are “dynamic”. For example, Als5p of *Candida albicans* reacts to mechanochemical pressure as it is stretched, evolving new conformations that propagate Als5p adhesive nanodomains on the fungal surface, while also drawing the host and pathogen together [32], [33]. By structural analysis, the repeats of BAD-1 are linked together by non-rigid “hinge” regions, like beads on a string. DLS analysis predicts an elongated conformation for BAD-1, arguing for a degree of flexibility in these hinge regions. If so, then a structure similar to the rod-like adhesins Als5p of *C. albicans* [34] and invasin of *Yersinia sp.* [35] is plausible as proposed in figure 3G.

Because the tandem repeats are necessary for virulence, we sought further insight into their structure and function. One notable aspect of their sequence is an exceptionally high tryptophan content. In an average protein perhaps one residue in a hundred will be tryptophan, but BAD-1 surpasses this ratio 17-fold. In nearly every repeat, three tryptophans are arranged in a highly conserved WxxWxxW pattern, a motif proven to mediate heparin binding by TSP-1. Short peptides bearing this motif are capable of binding to GAGs [22], and the presence of adjacent basic sequences enhances both affinity and specificity for heparin. In TSP-1, tryptophans are arrayed along a short, α-helical domain, coordinating with basic residues on an adjacent anti-parallel strand to create a surface-exposed recognition groove. The basic residues intercalate between the tryptophans, thus sharing their pi-orbital electrons in a configuration that is key for heparin association [36]. NMR analysis of the BAD-1 tandem repeat model - TR4 - demonstrated that the disulfide bond present within each repeat constrains the 17 residues between them into a consistent, tightly folded loop. While this loop localizes basic residues adjacent to the tryptophans of the WxxWxxW motif, one fold of this loop stretches transversely across these putative active residues in a manner that appears to interdict surface-exposure. In this way, the 3-D structure of TR4 contrasts with the heparin-binding motif of TSP-1. Yet, strong parallels between BAD-1 and TSP-1 prompted us to explore heparin-binding activity.

Our work discloses a previously unrecognized capacity of BAD-1 to bind heparin. The activity is saturable, specific and high-affinity, and constitutes an advance in our understanding of BAD-1 and *B. dermatitidis* pathogenesis. BAD-1 is known to alter host innate immune responses, suppressing TNF-α [2] and inducing TGF-β production [5]. We now show that BAD-1 suppresses T lymphocyte receptor signaling in a manner similar to that reported for TSP-1 [27]. TSP-1 binds to a heparan sulfate-modified serine residue of CD47, suppressing T cell activation. We do not provide direct biochemical evidence that BAD-1 binds CD-47, but our data show that BAD-1 similarly suppresses T cell function in a CD-47 dependent manner. This BAD-1 mimicry of TSP-1 resulted in impaired T cell activation and differentiation, with reduced production of effector cytokines including IL-17 and IFN-γ. Since T cell activation is vital to the host's ability to clear yeast via adaptive immunity [29], inhibition of T cell function could foster pathogen survival and immune evasion. TSP-1 also regulates immune-tolerance by phagocytic cells [37], nitric oxide signaling [38], activation of TGF-β [39], [40] and binding and clearance of matrix metalloproteinases involved in the healthy egress of lung inflammatory cells [41]. The ability of BAD-1 to mimic TSP-1 and modulate host immunity to its advantage could hinge on any or all of these activities.

Despite the evolutionary similarity between fungi and mammals, molecular mimicry is infrequently reported in pathogenic fungi. *Candida albicans* expresses an integrin-like protein Int1p [42] that binds yeast to vascular endothelium and promotes filamentation and virulence [43], [44]. *Histoplasma capsulatum* CBP1, a virulence factor, is similar in its 3-D NMR structure to mammalian saposin B [45], but evidence of saposin-like interactions with host glycolipid has not yet been reported. We describe a striking example of molecular mimicry involving sequences in the BAD-1 tandem repeat that mimic those in mammalian TSP-1 structurally and functionally, and confer pathogen survival.

There are additional implications stemming from the finding that BAD-1 binds heparin. Binding of mammalian cell surface GAGs is a mechanism used by many pathogens. Viruses, bacteria, and parasites exploit host cell-surface GAGs, mediating attachment with adhesins to impede clearance [46], [47]. The BAD-1 adhesin similarly binds yeast to lung tissue [48], macrophages [1], [6], [12] and ECM. While we do not provide evidence here that the adhesive features of BAD-1 are due to its affinity for heparin, parallels with other pathogens make this idea plausible.

Our observation that the TR4 repeats did not share the heparin-binding activity of BAD-1 was at first surprising because NMR analysis established structural identity between TR4 and the native BAD-1 repeats. Perhaps the need for an activation trigger in a critical pathogenicity factor like BAD-1 makes sense. Forty-one tandem repeats constitute enormous potential avidity, and non-specific adherence may disadvantage the pathogen. Given that a segment of the repeat loop lies across the putative heparin-binding motif, it was perhaps to be expected that our “model” tandem repeat would bind heparin poorly. Our observation that relaxing TR4's structure via disulfide reduction brings its heparin-binding up to parity with BAD-1 suggests that a reconfiguration of the loop structure may be requisite for heparin binding. Enzymatic reduction of disulfide bonds in the extra-cellular environment is one means of achieving this [49]. Plasmin is activated this way [50], and gp120 of HIV-1 requires the reduction of two disulfide bonds by cell-surface protein disulfide isomerase (PDI) to ligate lymphocyte receptors [51]. Importantly, reductases like PDI are apt to collect on cell surfaces enriched with GAGs [51].

There are other means of exchanging disulfide bonds. Cell-surface adhesins exposed to significant mechanochemical stress (stretching) are subject to accelerated disulfide cleavage and reorganization after initial ligation of target molecules [33], [52], [53], [54]. We hypothesize that BAD-1 associates initially with host cell-surface GAGs through its N-terminal Cardin-Weintraub domain [26], and that subsequent reorganization of its disulfide structure, either catalyzed or spontaneous, permits the tandem repeats to participate in heparin binding. This model would be expected to magnify the strength of the interaction (consistent with the slow dissociation rate of BAD-1 and heparin observed via SPR) and effectively draw the host and pathogen together as additional repeats are engaged, essentially “zippering” BAD-1 and yeast down onto heparin.

**Figure 8:**
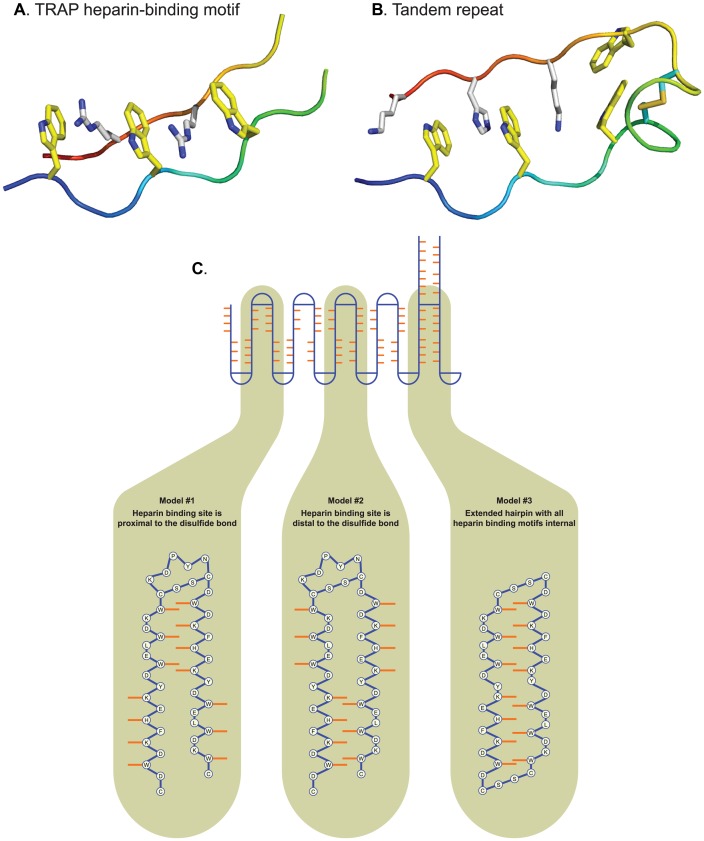
Theoretical model of tandem repeat heparin-binding domain. The thrombospondin-related anonymous protein (TRAP) of the malaria parasite from PDB (code 1LSL) was used as a template for modeling the backbone of theoretical models. **(A)** The 3-D structure of TRAP itself is a modified triple β sheet, with 3 tryptophans (yellow) on one strand buried internal to the triple sheet. The guanidinium groups from arginines (white) of the neighboring strand intercalate the tryptophans to form stacked pi clouds. The tryptophan strand is a distorted β sheet with the sequence WxxWxxW, while the intercalation chain is a β sheet with the sequence QxRxRx. **(B)** In the 3-D model presented for the BAD-1 tandem repeat, the intercalation between tryptophans (yellow) and basic residues (white) takes place “proximal” to the disulfide bond. Histidine and lysine residues replace the arginines present in the TRAP structure (nitrogens, blue). Theoretical structures were constructed and energy minimized in Sybyl. **(C)** Depiction in 2-dimensions of possible configurations of WxxWxxW tryptophans and BxBxB basic residues as they might intercalate to form a heparin-binding cleft in proximity to bonded cysteine residues (horizontal blue line). In the “proximal” (left) and “distal” (center) models, these interactions would likely stabilize a repeating, anti-parallel, β-sheet secondary structure. In the third “hairpin” model (right), tandem repeats would run in long, anti-parallel stretches to form extended hairpin structures.

Our effort to solve the NMR structure of TR4 complexed with heparin was unsuccessful. The 3-D structure of the tandem repeat motif that binds heparin therefore remains uncertain and is the subject of ongoing work. Figure 8 depicts a theoretical configuration of the tandem repeat structure (8B), showing the alignment of its residues in accordance with the heparin-binding motif of the thrombospondin-related anonymous protein (TRAP) of the malaria parasite (8A). In the “proximal” model (Fig. 8B and Fig. 8C, left image), the repeats would form a regular, anti-parallel β-sheet conformation. Alternative structures are possible including a so-called “distal” model also forming a β-sheet (Fig. 8C, middle) and a “hairpin” model (Fig. 8C); the latter two are rendered in 3-D in Figure S6.

In conclusion, we describe here structural features of BAD-1 tandem repeats, as determined by NMR, and a novel heparin-binding function associated with this essential virulence domain. This activity may govern *B. dermatitidis* yeast adherence to host cells and ECM via binding to heparan sulfate. In binding heparin, BAD-1 mimics TSP-1 in similarly down-regulating the activation of T cells through its interaction with a GAG-modified serine of CD47. These findings shed new light on the structure of BAD-1, its diverse functions, and novel mechanisms through which it may promote fungal pathogenicity. BAD-1 is a rare, but stunning example of molecular mimicry among fungal pathogens.

## Materials and Methods

### Ethics statement

All animal procedures were performed in accordance with the recommendations in the Guide for the Care and Use of Laboratory Animals of the National Institutes of Health. Care was taken to minimize animal suffering. The work was done with the approval of the IACUC of the University of Wisconsin-Madison.

### Reagents

Complete Mini protease inhibitor tablets (EDTA free) were from Roche (Indianapolis, IN). Heparin was from Sagant pharmaceuticals (Schaumberg, IL). Unless noted otherwise, chemicals were from Sigma.

### Fungi

American Type culture Collection (ATCC) strain 26199 of *B. dermatitidis*, a wild-type, virulent isolate originally obtained from a human patient, was used in this study, together with the isogenic attenuated, BAD-1 knockout strain #55 [48]. Truncated chimeras of the BAD-1 gene were used to transform strain #55 to secrete a full length BAD-1 with a 6-his tag (BAD1-6H), BAD-1 lacking the C-terminal region (ΔCterm as previously described) [12], and BAD-1 lacking 20 of the tandem repeats (Trepeat20)(described below). All isolates of *B. dermatitidis* were maintained in the yeast form on Middlebrook 7H10 agar slants with oleic acid-albumin complex, grown at 39°C. Liquid cultures of yeast were grown in *Histoplasma* macrophage media (HMM) [14].

### Expression of a truncated form of BAD-1, Trepeat20

An expression cassette for a truncated form of BAD-1 in which 20 of the tandem repeats were deleted was created by digesting the deletion construct pBAD1-6H [12] with BamH1 restriction enzyme (NEB Biolabs, Ipswich, MA) and then re-ligating, removing 1355 bp of the original cDNA. The deletion construct was excised from the pUC18 vector in an EcoR1/Xba1 digest, and then inserted into the EcoR1/Xba1 sites in the polylinker of plasmid pCB1532 (a vector carrying the Sulphonyl Urea resistance [SUR] gene of *Magneportha grisea*, generously provided by Dr. James Sweigard [Dupont, Wilmington, DE]) [55]. This plasmid was used to transform BAD-1 knockout strain #55 and producing strains were identified by Western blotting nitrocellulose overlays placed on replica plates of picked transformants, as previously described [48]. Strains were selected that most closely reconstituted the BAD-1 production seen in the 26199 parental strain. Production levels were estimated by Western blot of surface extracted protein probed with anti-BAD-1 mAb DD5-CB4 [1], [6] followed by goat anti-mouse (GAM) IgG-alkaline phosphatase (Promega). Production levels were further quantified using a FACscan flow cytometer (Becton Dickenson) using DD5-CB4 and GAM-FITC (Sigma) (Fig. S1).

### Murine model of *B. dermatitidis* infection

Male BALB/c mice ∼5–6 wk of age (Harlan Sprague Dawley) were infected intra-nasally with *B. dermatitidis* yeasts as previously described [56]. In brief, mice were anesthetized with inhaled Metafane (Mallinckrodt Veterinary Inc.). A 25-µl suspension of yeast cells in PBS was then applied drop-wise into their nares. To insure a lethal infection was established, 10^4^ yeast were thus administered. All animal procedures were performed in accordance with the recommendations in the Guide for the Care and Use of Laboratory Animals of the National Institutes of Health. Care was taken to minimize animal suffering. The work was done with the approval of the IACUC of the University of Wisconsin-Madison.

### Expression of recombinant 4-repeat model protein in *E. coli*

Complementary oligos RS33 and RS34 were annealed and ligated into pUC18 digested with BamH1/EcoR1 to make pUC18/33-34.

(RS33-GATCCGAAGACGACCCTACAACTGTGACTGGGACAAGTCCCATGAGAAGTATGATTGGGAGCTCTGGGATAAGTGGTGCAAGGACG,

RS34-AATTCGTCCTTGCACCACTTATCCCAGAGCTCCCAATCATACTTCTCATGGGACTTGTCCCAGTCACAGTTGTAGGGTCGTCTTCG)

pUC18/33-34 contains DNA coding for one tandem repeat, with a Bbs I site and a BamHI site just upstream. The annealed complementary oligos RS35 and RS36 were ligated into these sites, creating a new set of BbsI/BamHI sites for the next digestion/ligation cycle. After each cycle, the newly cloned plasmid was opened by Bbs I/BamHI and purified from an agarose gel (Freeze and Squeeze DNA extraction spin columns, Bio-Rad).

(RS35- GATCCGAAGACGACCCTACAACTGTGACTGGGACAGTCCCATGAGAAGTATGATTGGGAACTCTGGGATAAGTGGTGCAAGGAC,

RS36- AGGGGTCCTTGCACCACTTATCCCAGAGTTCCCAATCATACTTCTCATGGGACTTGTCCCAGTCACAGTTGTAGGGTCGTCTTCG)

This process was repeated until the plasmid contained four repeats. The final construct was cut out of pUC18 with BamH1/EcoRI (EcoR1 site blunted) and ligated into pQE32 cut with BamH1/HindIII (HindIII site blunted) creating plasmid pQETR4 for expression of the 4-repeat model protein in *E. coli* with a 6-histidine tag (TR4).

TR4 (Fig. 1C)

MRGSHHHHHHGIRRRPYNCDWDKSHEKYDWELWDKWCKDPYNCDWDKSHEKYDWELWDKWCKDPYNCDWDKSHEKYDWELWDKWCKDPYNCDWDKSHEKYDWELWDKWCKDELA

TR4 was expressed in *E.coli* (grown at 30°C in LB medium) by induction with IPTG and isolated from cell lysates using an NiNTA column (Qiagen, Valencia, CA). Protein was refolded while immobilized on NiNTA resin by subjecting it to a gradient from 100% buffer A (6M urea, 10 mM hepes, pH 8, 300 mM NaCl, 10% glycerol, 2 mM mercaptoethanol, 1 mM CaCl_2_) to 100% buffer B (10 mM hepes, pH 8, 300 mM NaCl, 10% glycerol, 5 mM 4∶1 GSH∶GSSG, 1 mM CaCl_2_) over the course of three hours. Refolded protein was then eluted with 250 mM Imidizole, which was removed by dialysis.

### Verification of disulfide linkages in TR4 by mass spectrometry

“In Liquid” digestion and mass spectrometric analysis was done at the Mass Spectrometry Facility (Biotechnology Center, University of Wisconsin-Madison). In short, 5 µg of purified protein in 125 mM NH_4_HCO_3_ (pH 8.5) was reduced with DTT (62.5 mM final) for 30 minutes at 55°C. Another 5 µg sample was left untreated as a control. Samples were spun through Pierce detergent removal columns (Thermo Scientific) to remove DTT and subsequently digested with trypsin solution (Trypsin Gold from Promega Corp.). Peptides were loaded on LC/MSD TOF (Agilent Technologies) and analyzed by MALDI TOF/TOF (AB SCIEX). (Additional detail available in supplementary data- Materials and Methods)

### Dynamic light scattering

The hydrodynamic radius of BAD-1 was measured by dynamic light scattering (DLS) collected at the 90 degree angle using a Beckman-Coulter N4 Plus instrument with the sampling time and prescaling as optimized by the instrument. Two samples of BAD-1 at 7 µM in 70 mM NaCl, 40 mM Tricine pH 7.0 were measured. For each sample ten autocorrelation functions were recorded and analyzed using both the unimodal and the size distribution software supplied with the instrument. The averages reported exclude repetitions with >1% baseline error in unimodal analysis or >5% dust fraction in the size distribution analysis. The refractive index of water at 15°C, 1.333, from instrument's database was used. The contribution of 40 mM tricine to the solvent viscosity was approximated by linear interpolation between increments reported for 20 and 100 mM tricine [57]. This contribution was added to the viscosity computed for 70 mM NaCl at 25°C, and then corrected to 15°C assuming its behavior paralleled that of water [58] for a value of 1.41 cP. The partial specific volume of BAD-1 was computed based on the sequence to be 0.704 mL/g. Hydration of 0.469 g water/g polypeptide at pH 7 was calculated based on the amino acid composition [59]. Additional detail is available in Supplementary Methods.

### Sedimentation equilibrium

Three samples of BAD-1 prepared by serial dilution were analyzed by sedimentation equilibrium to ascertain the association state. The buffer was 10 mM sodium phosphate, 100 mM NaCl at pH 7.6 with a computed density of 1.005 g/mL [47] and a partial specific volume of 0.704 mL/g. Equilibrium data at 4°C were collected using a Beckman analytical ultracentrifuge. Gradients were monitored at 280 nm and equilibrium data was recorded at speeds of 5600, 7800, 9200 and 12000 rpm. Analysis utilized software written in IGOR Pro (Wavemetrics, Inc.) by D. R. McCaslin. Additional detail is available in Supplementary Methods.

### Isolation of BAD-1

BAD-1 was purified as described [10] with a modification. Yeast was grown in liquid HMM in a gyratory shaker at 37°C for 5 days. Yeast was pelleted and washed once in PBS, and BAD-1 was released from cell surfaces with three 1-hour washes in dH_2_O and then purified on a metal-chelate resin (NiNTA). Due to its divalent cation-binding property, BAD-1 protein could be purified on NiNTA resin regardless of whether it included a 6-histidine tag. The stringency of the wash buffer was reduced by eliminating imidazole and reducing NaCl to 150 mM. Homogeneity of purified BAD-1 was analyzed by SDS-PAGE, Sypro Ruby stain (Invitrogen), and Western blot using anti-BAD-1 antibody (DD5-CB4) [1], [6], [60].

### Binding of yeast to Matrigel

Matrigel (Collaborative Biomedical Products, Bedford, MA) was diluted to 5 mg/ml, 0.5 mg/ml and 0.05 mg/ml in RPMI. 30 µl was put into wells of a 96-well plate and allowed to gel at 37°C overnight. *Blastomyces* yeast were labeled with Na^51^CrO_4_ for 105 minutes at 37°C, washed and diluted to 2×10^7^/ml in HBSS+0.1%BSA. 50 µl was added to each well and incubated for 60 minutes, then washed with HBSS. Well contents were subjected to scintillation counting (compared to controls with known numbers of labeled yeast) to quantify bound yeast. To block BAD-1-mediated binding, rabbit anti-BAD-1 immune serum [8] (or control pre-immune serum) was applied to labeled yeast, incubated for 1 hour, and washed with HBSS before binding assays.

### Native BAD-1 binding to heparin-agarose

Heparin-agarose resin was obtained from Sigma as were control agarose beads and agarose beads coated with BSA, hemoglobin, and mannan. 100 µl of 0.1 mg/ml BAD-1 was incubated with agarose resins (5 µl bed volume) in 20 mM tricine, pH 7, 50 mM NaCl for 30 min at 25°C with agitation. Resin beads were pelleted by centrifugation in a microfuge at 7000xG. Concentration of BAD-1 before and after incubation with resin was monitored by A280 via Nanodrop Spectrophotometer (ND1000, Thermo Scientific). Binding was calculated by comparing the A280 of supernates to that of starting material. Binding inhibition studies were done with soluble medical grade heparin purchased from Elkins-Sinn Inc (Cherry Hill, NJ), dermatan sulfate (chondroitin sulfate B)(Sigma), chondroitin sulfate A (chondroitin-4-sulfate, fraction A)(Sigma) and hyaluronan (Sigma). Baseline absorbance was corrected to account for absorbance of added GAG inhibitors. During optimization studies, heparin resin bed volume was varied from 1 µl to 20 µl, NaCl concentrations were varied from 40 mM to 2000 mM and alternative buffers were tested (20 mM Na-acetate, pH 5, 20 mM Tricine pH 7 and pH 8, 20 mM Na-carbonate, pH 9). Reduction of BAD-1 and TR4 was accomplished with 10 mM DTT and heating at 100°C for three minutes. Re-oxidation of thiols was accomplished by diluting or dialyzing away DTT followed by exposure to air at room temperature.

### Fluorescent BAD-1 binding to heparin-agarose

BAD-1 binding to heparin and control resins, and binding inhibition studies also were performed using BAD-1 labeled with the eFluor605NC kit from eBioscience (San Diego, CA). BAD-1 was fluorescently labeled following the manufacturer's instructions. BAD-1 (eFluor605) was incubated with heparin-coated agarose beads or control beads in 20 mM tricine buffer, pH 7, 50 mM NaCl and washed three times with the same buffer before quantification. Association of BAD-1 (eFluor605) with resins was verified visually using an Olympus BX60 fluorescent microscope. For binding and inhibition studies, BAD-1 (eFluor605) was quantitated using a FilterMax F5 multi-mode microplate reader (Molecular Devices, Sunnyvale, CA) and opaque 96 well plates (Costar, Cambridge, MA). BAD-1 binding to heparin also was reproduced at higher ionic strengths of 100 mM NaCl and 150 mM NaCl, as observed in alveolar mucus and plasma, respectively [16], [17].

### Production of isotopically-labeled TR4 and native BAD-1 for NMR analysis

*E. coli* strain XL-1 Blue transformed with pQE-TR4 was grown in defined M9 medium supplemented with ^15^N ammonium chloride (1 g/L) and ^13^C dextrose (4 g/L) (Cambridge Isotope Laboratories Inc., Andover, MA) for 20 hrs at 30°C, under ampicillin selection (100 µg/ml) followed by induction with IPTG and 4 hours of further incubation. [^13^C,^15^N]-labeled TR4 was purified and refolded as described above. ^15^N labeled native BAD-1 was produced by growing 26199 *B. dermatitidis* yeast at 37°C for five days in M9 medium supplemented with ^15^N ammonium chloride (1 g/L) and purifying as described above. Purity of isolated proteins was verified by PAGE prior to NMR analysis.

### NMR spectroscopy

All NMR spectra were recorded at the National Magnetic Resonance Facility at Madison (NMRFAM) on Varian VNMRS (600 MHz and 900 MHz) spectrometers equipped with triple-resonance cryogenic probes. The temperature of the sample was regulated at 25°C for experiments. For sequence specific backbone resonance assignments, a series of two-dimensional (2D) and three-dimensional (3D) heteronuclear NMR spectra were collected on a sample containing 0.1 mM of the [^13^C,^15^N]-labeled TR4 dissolved in NMR buffer with 10 mM phosphate, pH 8.0, 95% H_2_O, 5% D_2_O [61]. Raw NMR data was processed with NMRPipe [62] and analyzed using the program Sparky [63]. 2D ^1^H-^15^N HSQC and 3D HNCO data sets were used to identify the number of spin systems, and these identifications plus 3D HNCACB and 3D CBCA(CO)NH data sets were used as input to the PINE server to determine sequence specific backbone resonance assignments [64]. Due to the complexity of the system, the automated backbone resonance assignments needed to be refined manually with the help of a 3D ^15^N-edited ^1^H-^1^H NOESY spectrum. To assign side chain and HB and HA resonances 2D ^1^H-^13^C aliphatic HSQC, 3D HBHA(CO)NH, 3D HC(CO)NH, 3D C(CO)NH and 3D H(C)CH TOCSY experiments were used. Furthermore, a 2D ^1^H-^13^C aromatic HSQC spectrum together with a 3D ^13^C aromatic-edited ^1^H-^1^H NOESY were used to assign resonances from aromatic side chains. Finally, a 3D ^15^N-edited ^1^H-^1^H NOESY (100 ms mixing time) spectrum, a 3D ^13^C aliphatic-edited ^1^H-^1^H NOESY (100 ms) spectrum and a 3D ^13^C aromatic-edited ^1^H-^1^H NOESY (100 ms) spectrum were acquired and used to derive the distance constraints to determine the three dimensional structure of the protein.

### Structure calculation and analysis

^15^N resolved ^1^H-^1^H 3D NOESY and ^13^C resolved ^1^H-^1^H 3D NOESY spectra were used to derive ^1^H-^1^H distance restraints. Backbone dihedral angle restraints φ and ψ were obtained from ^1^H, ^15^N, ^13^CA, ^13^CB, ^13^C′ using TALOS+ software [65]. CYANA software version 3.0 was used for automated NOESY peaks assignments and structure calculation following the standard simulated annealing protocol [66]. The program PYMOL (Schrödinger sales) was used to calculate the root mean square deviation (rmsd) and for graphical analysis. The PSVS server was used to check the quality of the structures [67].

### Surface plasmon resonance

A Biorad Proteon XPR36 instrument was used for surface plasmon resonance studies. To prepare a heparin surface, medical grade heparin was biotinylated with EZ-link Biocytin Hydrazide (Thermo Scientific) and 1-Ethyl-3-(3-dimethylaminopropyl) carbodiimide (EDAC) following manufacturer's instructions. Unreacted biotin was removed by precipitating biotinylated heparin with 66% methanol, which was then applied in binding buffer (50 mM Hepes, pH 7, 70 mM NaCl, 0.1% Tween-20) (0.1 mg/ml) to a Biorad neutravidin (Proteon Sensor Chip NLC) according to the manufacturer's protocol. Subsequently, single and multiple injections of varying lengths of time and with varied concentrations of biotinylated-heparin were made onto chip in its vertical orientation. After immobilization, the surface was washed with several injections of 1 M NaCl in 50 mM NaOH. Final immobilization levels of biotinylated heparin were: 0, 5, 30, 59 and 96 RU. Analysis of BAD-1 binding utilized the horizontal orientation of the chip; length of injection and dissociation time varied, but the flow rate was a constant 30 µL/min. BAD-1 remaining bound at the end of the dissociation period was removed by a 1-minute pulse at 30 µL/min of 1 M NaCl, 50 mM NaOH. The buffer used in the binding studies was 20 mM Hepes pH 7,100 mM NaCl, and 0.1% Tween20. To demonstrate specificity of the binding, a mixture of BAD-1 and heparin were co-injected across the surfaces.

Baseline and injection alignments used software supplied with the instrument. BAD-1 did bind to surfaces lacking heparin, but to a much lesser degree than to modified surfaces; therefore, the inter-spot data was used as a reference to correct for this non-specific binding. The kinetic analysis of the corrected sensorgrams utilized the 1∶1 Langmuir binding model in the software. The fitted curves shown in Figure 5A allowed the on and off rates for each trace to vary but were constrained to a common value for each heparin density.

### Synthesis of peptide competitors for heparin binding

The UW-biotechnology center synthesized a peptide of SHWSPWSS based on the published sequence known to bind to heparin and inhibit binding by TSP-1 [15]. This peptide represented a minimal version of the WxxWxxW heparin-binding motif. A control peptide SHQSPQSS was synthesized in which the tryptophans were replaced with glutamine residues. These peptides were analyzed for purity by HPLC and mass spec and purified/desalted chromatographically.

### Pre-treatment of heparin resin with binding competitors

Heparin resin was washed with binding buffer (20 mM tricine buffer, pH 7, 50 mM NaCl) three times. Binding competitors (WxxW peptide, control peptide, TR4 or reduced TR4) were added to 4 cubic mm of resin and incubated with agitation at room temp for 20 min. Resin was washed once with binding buffer before addition of fluorescent BAD-1 (eFluor605) and incubation continued for another 20 min. Resin was washed three times with binding buffer and binding was quantified by fluorescence on a FilterMax F5 multi-mode microplate reader as above.

### T-cell inhibition

T cell inhibition was assessed as described with minor modifications [27]. Anti-CD3 antibody (5 µg/mL) was immobilized on Nunc Maxisorp 96-well round bottom plates in carbonate buffer (pH 9.5) for 1 hr. Parental Jurkat or related JinB8 (CD47-deficient) T cells were pre-incubated for 10 min at 37°C with 10 µg/mL of recombinant TSP-1 (R&D Systems) or native BAD-1 prior to activation with immobilized anti-CD3 antibody for 2 hr. Total RNA was isolated using an RNeasy kit (Qiagen) and RNA was reverse transcribed using iScript cDNA synthesis kit following manufacturer's instructions (Bio-Rad). Real-time PCR primers for human CD69 and human HPRT1 were generated as described [27]. Real-time PCR was performed using SsoFast EvaGreen Supermix (Bio-Rad) on a MyIQ real-time PCR detection system (Bio-Rad). Fold change in CD69 mRNA expression was normalized to HRPT1 mRNA levels.

Primary T cells were obtained from *Blastomyces*-reactive 1807 TCR transgenic mice [30]. CD4^+^ T cells were purified with magnetic beads (BD Biosciences, Franklin Lakes, NJ) according to the manufacturer's instructions. Purified 1807 cells (3×10^5^/well) were added to co-cultures of *B. dermatitidis* yeast strain #55 (3×10^5^/well) and bone-marrow derived DCs (3×10^5^/well). After 96 hours of co-culture, supernate was harvested and tested for levels of IL-17A or IFN-γ

according to manufacturer's instructions (R&D Systems, Minneapolis, MN), and T cells were analyzed by FACscan flow cytometry (BD Biosciences) for activation as measured by surface display of CD69, CD25, CD44, and CD62L (eBioscience, San Diego, CA; and BD Biosciences). 1807 cells were detected with antibody against the surface Thy1.1 marker specific for the T cells. In some experiments of T cell suppression, 1807 cells were pre-incubated with BAD-1 in varied amounts in PBS/0.5% BSA for 90 minutes at 37°C, and the cells were washed to remove free BAD-1 before addition into the assay. BAD-1 was also tested for suppression of T cell function by adding the protein directly into the co-culture of yeast, DC and T cells.

### CD47 transfection

CD47-deficient JinB8 T cells were transiently transfected with plasmids encoding either CD47 or CD47 with a serine-to-alanine mutation at position 64 (CD47-S64A)(a generous gift of Dr. David Roberts) using Lipofectamine Plus (Invitrogen). Transfections were performed overnight prior to initiation of experiments. To verify re-expression, untransfected and transfected JinB8 cells were incubated with PE-conjugated anti-CD47 antibody (BD Bioscience) and analyzed by flow cytometry. T cell inhibition studies were done, as above, using these transfected cells.

### Theoretical modeling of the conformation of heparin-binding tandem repeats

The disulfide loop was created using the Rosetta suite of protein structure prediction software (www.rosettacommons.org). The sequence WCKDPYNCD produced several models of the disulfide region, with the best-fit model used to link the two chains. All the models were energy minimized using the Sybyl suite of Tripos software and the Tripos force field until convergence. All modeling was performed in Sybyl.

### Statistical analysis

Kaplan Meier [68] survival curves were generated for mice that received a lethal infection. Survival times of mice that were alive by the end of the study were regarded as censored. Time data were analyzed by the log rank statistic and exact *P* values were computed using the statistical package Stat Xact-3 by CYTEL Software Corporation. Survival of different groups are considered significantly different if the two-sided *P* value is <0.05. When multiple comparisons were made simultaneously, *P* values were adjusted according to Bonferroni's correction to protect the overall significance level of 0.05. All binding data was analyzed by Prism (Graphpad Corp.) with error bars representing simple SEM.

### Accession number

The coordinates and structure factors have been deposited at the Protein Data Bank (PDB) with the following accession codes. PDB: 2LWP; BMRB: 18618.

### Supplementary data

Supplementary Methods and Figures are available and appended.

## Supporting Information

Figure S1 **Recombinant** ***B. dermatitidis*** **yeast displaying BAD-1 derivatives.** Yeast were probed with primary anti-BAD-1 monocolonal antibody DD5-CB4 and a secondary GAM-FITC antibody (Sigma) to quantify surface BAD-1 using a FACscan flow cytometer (Becton Dickenson). *B. dermatitidis* yeast transformed to produce truncated forms of BAD-1 (Trepeat Y and –AE, bearing half the normal number of tandem repeats) displayed as much or more BAD-1 on their surfaces as yeast transformed to produce full-length BAD-1 (BAD1-6H J and –AC). Truncated and full-length forms both included a 6-histidine tag for purification. MFI = mean fluorescence intensity.
(EPS)

Figure S2 **Refolding and analysis of TR4 expressed and purified from** ***E.coli*** **.** TR4 migrated at varied *Mr* under non-reducing conditions (Non-reduced), and chiefly at 10 kD under reducing conditions (Reduced TR4). Refolding conditions and glutathione gradient parameters were adjusted until TR4 eluted from the NiNTA column as a single predominant band (Refolded). This band migrated at 14 kD and was subjected to NMR analysis to confirm that the residues in refolded TR4 and the residues of native BAD-1 existed in identical environments.
(EPS)

Figure S3 **BAD-1 binding to heparin agarose BAD-1 Heparin binding was quantified by comparing the initial A280 of the purified, soluble BAD-1 to the A280 of BAD-1 in the unbound aqueous phase. (A)** Binding of BAD-1 to heparin and alternative resins. Heparin agarose resin pulled down the majority of BAD-1 in this assay (right column). The relative binding of BAD-1 to no-agarose control, uncoated agarose resin and resins coated with BSA, hemoglobin, ConA and mannan was measured for comparison. BAD-1 bound better to heparin agarose than control resins (*, p<0.05) and binding to control resins was insignificant (p>0.05). **(B)** Inhibition of BAD-1 binding to heparin resin by soluble heparin. BAD-1 was pre-incubated with increasing amounts of soluble heparin prior to exposure to heparin-agarose resin. **(C)** Inhibition of BAD-1 binding to heparin agarose by alternate GAGs. 0.1 mg/ml BAD-1was pre-incubated with heparin, chondroitin sulfate A, or hyaluronan for 20 min, followed by exposure to heparin-agarose for an additional 30 min. The A280 of the starting BAD-1 solution was 0.8±0.04 and the A280 of the positive binding control was 0.4±0.04 (∼50% binding). Heparin inhibited binding significantly better than controls (*, p<0.05), while inhibition by chondroitin and hyaluronan **were not significant** (p>0.05).
(EPS)

Figure S4 **SPR of BAD-1 lacking 20 copies of the tandem repeat (TrepeatΔ20).** Interaction of TrepeatΔ20 with heparin measured by surface plasmon resonance (SPR). TrepeatΔ20 binding was monitored using a Biorad Proteon XPR36. TrepeatΔ20 at the indicated concentrations was injected onto Biorad NLC neutravidin surface with biotinylated heparin immobilized to levels of 5 (circles) and 30 (squares) RUs. For clarity, only every 15th data point is shown. The solid lines are fits to the Langmuir binding model. On and off rates were fit to each sensogram, but maximal response was fit to a single value for each immobilization level.
(EPS)

Figure S5 **Effect of salt and pH on binding of BAD-1 to heparin.** Effect of NaCl **(A)** and pH **(B)** on binding of BAD-1 to immobilized heparin. A known concentration of BAD-1 was incubated with heparin agarose. The percentage of BAD-1 bound was quantified by measuring A280 of supernate after incubation, compared to that of the starting material. Results are the mean ± SEM of two independent experiments.
(EPS)

Figure S6 **3-D representation of a model of the tandem repeat heparin-binding domain. (A)** The “distal conformation” has the tryptophans of the WxxWxxW motif intercalating with the basic residues of the BxBxB motif distal to the disulfide bond, in contrast to the proximal model. **(B)** In the “hairpin conformation”, the tryptophans and basic residues intercalate both proximally and distally to the disulfide bond, but instead of forming a repeating, anti-parallel β-sheet, repeats in this configuration would be expected to form extended hairpin structures. Please see Figure 8B for an illustration of the “proximal conformation”.
(EPS)

Text S1 **Supplementary Materials.**
(DOCX)
