# Supplementary material for: Structure and Function of a Fungal Adhesin that Binds Heparin and Mimics Thrombospondin-1 by Blocking T Cell Activation and Effector Function
Source: PLoS Pathog. 2013 Jul 11;9(7):e1003464. doi: 10.1371/journal.ppat.1003464 (PMC3708853; doi:10.1371/journal.ppat.1003464)
Supplement: Text S1 — Supplementary Materials. (DOCX) [file ppat.1003464.s007.docx]

**SUPPLEMENTARY MATERIAL**

**METHODS**

**Analysis of disulfide binding in TR4**

“In Liquid” digestion and mass spectrometric analysis was done at the Mass Spectrometry Facility [Biotechnology Center, University of Wisconsin-Madison]. In short, 5µg [15µl] of purified protein in 125mM NH_4_HCO_3_ [pH 8.5] was reduced with 5ul of 250mM DTT [62.5mM final] for 30 minutes at 55⁰C, and another 5µg sample was left on ice in its native state as a control. Post reduction, both treated and control samples were spun through Pierce detergent removal columns [Thermo Scientific] to remove the majority of DTT from the reduced sample. Samples were subsequently denatured with 10μl of 8M Urea and diluted to 50μl for tryptic digestion with: 2.5μl ACN, 7.5μl of 0.2% ProteaseMAX™ (Promega Corp.), 5μl 25mM NH_4_HCO_3_ (pH 8.5) and 5μl trypsin solution (10ng/μl *Trypsin Gold* from PROMEGA Corp. in 25mM NH_4_HCO_3_). Digestion was conducted for 60 minutes at 40°C, then an additional 2.5µl of trypsin solution was added (final enzyme:substrate 1:67) and digestion proceeded overnight at 37°C. The reaction was terminated by acidification with 2.5% TFA (Trifluoroacetic Acid) to 0.3% final. Degraded ProteaseMAX™ was removed via centrifugation (max speed, 10minutes) and the peptides loaded directly on LC/MSD TOF (Agilent Technologies) or solid phase extracted (*ZipTip^®^ C18* pipette tips, Millipore) and analyzed on MALDI TOF/TOF (AB SCIEX).

**MALDI TOF/TOF Analysis**

Peptides were eluted off the C18 column with 1ul of acetonitrile/H_2_O/TFA (60%:40%:0.1%) into 0.5ml Protein LoBind tube (Eppendorf), and 0.5ul was deposited onto the Opti-TOF™ 384 Well plate (AB SCIEX) and re-crystalized with 0.5µl of matrix (10mg/ml α-Cyano-4hydroxycinnamic acid in acetonitrile/H_2_O/TFA [75%:25%:0.1%]). Peptide Map Fingerprint result-dependent MS/MS analysis was performed on a 4800 Matrix-Assisted Laser Desorption/Ionization-Time of Flight-Time of Flight (MALDI TOF-TOF) mass spectrometer (AB SCIEX). In short, peptide fingerprint was generated scanning 700-4,000 Da mass range using 1000 shots acquired from 20 randomized regions of the sample spot at 4000 intensity of OptiBeam™ on-axis Nd:YAG laser with 200Hz firing rate and 3 to 7ns pulse width in positive reflectron mode. Fifteen most abundant precursors, excluding trypsin autolysis peptides and sodium/potassium adducts, were selected for subsequent tandem MS analysis where 1200 total shots were taken with 4400 laser intensity and 1.5 kV collision induced dissociation (CID) using air. Post-source decay (PSD) fragments from the precursors of interest were isolated by timed-ion selection and reaccelerated into the reflectron to generate the MS/MS spectrum. Raw data was deconvoluted using GPS Explorer™ software and submitted for peptide mapping and MS/MS ion search analysis against user defined *Blastomyces dermatitidis* database (19,127 protein entries) with an in-house licensed Mascot search engine ([Matrix](http://www.matrixscience.com) Science, London, UK) with Methionine oxidation and Lysine carbamylation as variable modifications.

***LC-MS analysis***

| Time (min) | %B |
| --- | --- |
| 0 | 2 |
| 1 | 2 |
| 25 | 90 |
| 26 | 95 |
| 27 | 2 |
| 42 | 2 |

Peptides and intact protein species were chromatographically resolved with Phenomenex Jupiter 5µm C4 300A 2.0x50mm column, equipped with guard column containing the same resolving media, on an Agilent 1200 HPLC with an autosampler held at 5⁰C, using linear gradient of 98% Water: 2% acetonitrile 0.1% Formic acid to 90% Acetonitrile 10% water 0.1% Formic acid over 25 minutes. The flow rate was .25 ml/min. The gradient was as follows:

The mass spectrometer used in conjunction with chromatographic separation was an Agilent LC/MSD TOF with electrospray ionization used in positive ion mode (<http://www.biotech.wisc.edu/facilities/massspec/instrumentationoverview/LCMSDTOF>)

Lists of peptide masses were generated in Agilent MassHunter Qualitative Analysis using the Find Compound by Molecular Feature. To deconvolute intact MW of protein species Agilent BioConfirm Software version A.02.00 was used. The following instrumental parameters were used to generate the most optimum protonated ions [M+H^⊕^] in Positive Mode: Capillary voltage 3600 V; Drying Gas 6.0 l/min; Nebulizer 30 psig; Gas temperature 350°C; Oct DC1 39.5 V; Fragmentor 130 V; Oct RF 250 V; Skimmer 60V. Internal calibration was achieved with assisted spray of two reference masses, 922.0098 m/z and 121.0509 m/z.

**DLS analysis of BAD-1**

Unimodal analysis of the dynamic light scattering (DLS) data from two independent samples of BAD-1 indicated a high level of polydispersity, 0.66 and 0.79. For both sets of data, the software identified a population accounting for ~70% of the scattering intensity with a mean radius of 7.2 ± 0.5 nm and mean standard deviation for the peak of 1.8± 0.7. The remaining scattering intensity was attributed to a population with a much broader size range. The mean radius among all repetitions was 111± 89 nm with a mean peak standard deviation of 16±16 nm. Interestingly the first peak was the same in the two protein samples, but the second peaks mean radius was very different 160 ± 22 and 68 ± 10. This analysis makes it clear that the BAD-1 preparations have some heterogeneity in size, possibly due to aggregation, but has a dominant fraction of a fairly homogeneous size of 7.2± 0.5 nm, which is taken to be the monomer.

**Sedimentation equilibrium analysis**

~100 µL of each sample was loaded into one sector of 1.2 cm charcoal-filled epon double sector centerpieces with ~108 µL of the dialysate buffer in the reference sector. Data were collected at 4°C with gradients monitored at a nominal 280 nm (the instrument reported values from 279-281 nm). Equilibrium was assumed when gradients collected 3 or more hours apart were superimposable. Equilibrium data were collected at speeds of 5600, 7800, 9200 and 12000 rpm. The presence of non-sedimenting baseline (BOD) was established by a high-speed spin to deplete all protein material. Loading absorbances (280 nm) were 0.636, 0.350 0.182 with BODs of 0.036, 0.022, and 0.013 respectively. Plots of the ln of the absorbance vs. squared distance from the center of rotation were linear at all speeds and for all loading concentrations consistent with a single molecular species, which was found to be monomeric. No evidence was found for higher oligomers up to at least up to a concentration of ~1.5 µM BAD-1.

**SPR analysis of BAD-1**

In a dose-response curve generated before the lowest density heparin surface was added, the off rates were comparable, but the on rates for all surfaces were generally higher, about twice the results discussed above, and maximal binding was higher. The source of this variation is unclear; it could reflect a change in the surface during removal of bound BAD-1, variability in the quality of BAD-1 preparation (degraded preparations show weak binding) since on rates are dependent on accurate concentrations or some combination of the two. The lower variation in off rates probably reflects the low degree of dissociation occurring during the measurements (half-life for dissociation of 1.1 h) and it is not as dependent on accurate concentrations. Nonetheless, BAD-1 clearly binds to immobilized heparin in a concentration dependent manner, which is inhibitable by free heparin, and once bound BAD-1 dissociates from the surface slowly.

**Figure S1.** **Recombinant *B. dermatitidis* yeast displaying BAD-1 derivatives**.

Yeast were probed with primary anti-BAD-1 monocolonal antibody DD5-CB4 and a secondary GAM-FITC antibody (Sigma) to quantify surface BAD-1 using a FACscan flow cytometer (Becton Dickenson). *B. dermatitidis* yeast transformed to produce truncated forms of BAD-1 (Trepeat Y and –AE, bearing half the normal number of tandem repeats) displayed as much or more BAD-1 on their surfaces as yeast transformed to produce full-length BAD-1 (BAD1-6H J and –AC). Truncated and full-length forms both included a 6-histidine tag for purification. MFI = mean fluorescence intensity.

**Figure S2. Refolding and analysis of TR4 expressed and purified from *E.coli.***

TR4 migrated at varied *Mr* under non-reducing conditions (Non-reduced), and chiefly at 10kD under reducing conditions (Reduced TR4). Refolding conditions and glutathione gradient parameters were adjusted until TR4 eluted from the NiNTA column as a single predominant band (Refolded). This band migrated at 14kD and was subjected to NMR analysis to confirm that the residues in refolded TR4 and the residues of native BAD-1 existed in identical environments.

Figure S3. BAD-1 binding to heparin agarose.

BAD-1 heparin binding was quantified by comparing the initial A280 of the purified, soluble BAD-1 to the A280 of BAD-1 in the unbound aqueous phase. (**A)** Binding of BAD-1 to heparin and alternative resins. Heparin agarose resin pulled down the majority of BAD-1 in this assay (right column). The relative binding of BAD-1 to no-agarose control, uncoated agarose resin and resins coated with BSA, hemoglobin, ConA and mannan was measured for comparison. BAD-1 bound better to heparin agarose than control resins (*, p<0.05) and binding to control resins was insignificant (p>0.05). **(B)** Inhibition of BAD-1 binding to heparin resin by soluble heparin. BAD-1 was pre-incubated with increasing amounts of soluble heparin prior to exposure to heparin-agarose resin. (**C)** Inhibition of BAD-1 binding to heparin agarose by alternate GAGs. 0.1mg/ml BAD-1was pre-incubated with heparin, chondroitin sulfate A, or hyaluronan for 20 min, followed by exposure to heparin-agarose for an additional 30 min. The A280 of the starting BAD-1 solution was 0.8 ± 0.04 and the A280 of the positive binding control was 0.4 ± 0.04 (~50% binding). Heparin inhibited binding significantly better than controls (*, p<0.05), while inhibition by chondroitin and hyaluronan were not significant (p>0.05).

**Figure S4. SPR of BAD-1 lacking 20 copies of the tandem repeat (TrepeatΔ20).**

Interaction of TrepeatΔ20 with heparin measured by surface plasmon resonance (SPR). TrepeatΔ20 binding was monitored using a Biorad Proteon XPR36. TrepeatΔ20 at the indicated concentrations was injected onto Biorad NLC neutravidin surface with biotinylated heparin immobilized to levels of 5 (circles) and 30 (squares) RUs. For clarity, only every 15th data point is shown. The solid lines are fits to the Langmuir binding model. On and off rates were fit to each sensogram, but maximal response was fit to a single value for each immobilization level.

**Figure S5. Effect of salt and pH on binding of BAD-1 to heparin.**

Effect of NaCl (**A)** and pH **(B)** on binding of BAD-1 to immobilized heparin. A known concentration of BAD-1 was incubated with heparin agarose. The percentage of BAD-1 bound was quantified by measuring A280 of supernate after incubation, compared to that of the starting material. Results are the mean ± SEM of two independent experiments.

**Figure S6**. **3-D representation of a model of the tandem repeat heparin-binding domain.** **(A)** The “distal conformation” has the tryptophans of the WxxWxxW motif intercalating with the basic residues of the BxBxB motif distal to the disulfide bond, in contrast to the proximal model. **(B)** In the “hairpin conformation”, the tryptophans and basic residues intercalate both proximally and distally to the disulfide bond, but instead of forming a repeating, anti-parallel β-sheet, repeats in this configuration would be expected to form extended hairpin structures. Please see Figure 8B for an illustration of the “proximal conformation”.
